# Sequence data and association statistics from 12,940 type 2 diabetes cases and controls

**DOI:** 10.1038/sdata.2017.179

**Published:** 2017-12-19

**Authors:** Flannick Jason, Christian Fuchsberger, Anubha Mahajan, Tanya M. Teslovich, Vineeta Agarwala, Kyle J. Gaulton, Lizz Caulkins, Ryan Koesterer, Clement Ma, Loukas Moutsianas, Davis J. McCarthy, Manuel A. Rivas, John R. B. Perry, Xueling Sim, Thomas W. Blackwell, Neil R. Robertson, N William Rayner, Pablo Cingolani, Adam E. Locke, Juan Fernandez Tajes, Heather M. Highland, Josee Dupuis, Peter S. Chines, Cecilia M. Lindgren, Christopher Hartl, Anne U. Jackson, Han Chen, Jeroen R. Huyghe, Martijn van de Bunt, Richard D. Pearson, Ashish Kumar, Martina Müller-Nurasyid, Niels Grarup, Heather M. Stringham, Eric R. Gamazon, Jaehoon Lee, Yuhui Chen, Robert A. Scott, Jennifer E. Below, Peng Chen, Jinyan Huang, Min Jin Go, Michael L. Stitzel, Dorota Pasko, Stephen C. J. Parker, Tibor V. Varga, Todd Green, Nicola L. Beer, Aaron G. Day-Williams, Teresa Ferreira, Tasha Fingerlin, Momoko Horikoshi, Cheng Hu, Iksoo Huh, Mohammad Kamran Ikram, Bong-Jo Kim, Yongkang Kim, Young Jin Kim, Min-Seok Kwon, Juyoung Lee, Selyeong Lee, Keng-Han Lin, Taylor J. Maxwell, Yoshihiko Nagai, Xu Wang, Ryan P. Welch, Joon Yoon, Weihua Zhang, Nir Barzilai, Benjamin F. Voight, Bok-Ghee Han, Christopher P. Jenkinson, Teemu Kuulasmaa, Johanna Kuusisto, Alisa Manning, Maggie C. Y. Ng, Nicholette D. Palmer, Beverley Balkau, Alena Stančáková, Hanna E. Abboud, Heiner Boeing, Vilmantas Giedraitis, Dorairaj Prabhakaran, Omri Gottesman, James Scott, Jason Carey, Phoenix Kwan, George Grant, Joshua D. Smith, Benjamin M. Neale, Shaun Purcell, Adam S. Butterworth, Joanna M. M. Howson, Heung Man Lee, Yingchang Lu, Soo-Heon Kwak, Wei Zhao, John Danesh, Vincent K. L. Lam, Kyong Soo Park, Danish Saleheen, Wing Yee So, Claudia H. T. Tam, Uzma Afzal, David Aguilar, Rector Arya, Tin Aung, Edmund Chan, Carmen Navarro, Ching-Yu Cheng, Domenico Palli, Adolfo Correa, Joanne E. Curran, Dennis Rybin, Vidya S. Farook, Sharon P. Fowler, Barry I. Freedman, Michael Griswold, Daniel Esten Hale, Pamela J. Hicks, Chiea-Chuen Khor, Satish Kumar, Benjamin Lehne, Dorothée Thuillier, Wei Yen Lim, Jianjun Liu, Marie Loh, Solomon K. Musani, Sobha Puppala, William R. Scott, Loïc Yengo, Sian-Tsung Tan, Herman A. Taylor, Farook Thameem, Gregory Wilson, Tien Yin Wong, Pål Rasmus Njølstad, Jonathan C. Levy, Massimo Mangino, Lori L. Bonnycastle, Thomas Schwarzmayr, João Fadista, Gabriela L. Surdulescu, Christian Herder, Christopher J. Groves, Thomas Wieland, Jette Bork-Jensen, Ivan Brandslund, Cramer Christensen, Heikki A. Koistinen, Alex S. F. Doney, Leena Kinnunen, Tõnu Esko, Andrew J. Farmer, Liisa Hakaste, Dylan Hodgkiss, Jasmina Kravic, Valeri Lyssenko, Mette Hollensted, Marit E. Jørgensen, Torben Jørgensen, Claes Ladenvall, Johanne Marie Justesen, Annemari Käräjämäki, Jennifer Kriebel, Wolfgang Rathmann, Lars Lannfelt, Torsten Lauritzen, Narisu Narisu, Allan Linneberg, Olle Melander, Lili Milani, Matt Neville, Marju Orho-Melander, Lu Qi, Qibin Qi, Michael Roden, Olov Rolandsson, Amy Swift, Anders H. Rosengren, Kathleen Stirrups, Andrew R. Wood, Evelin Mihailov, Christine Blancher, Mauricio O. Carneiro, Jared Maguire, Ryan Poplin, Khalid Shakir, Timothy Fennell, Mark DePristo, Martin Hrabé de Angelis, Panos Deloukas, Anette P. Gjesing, Goo Jun, Peter Nilsson, Jacquelyn Murphy, Robert Onofrio, Barbara Thorand, Torben Hansen, Christa Meisinger, Frank B. Hu, Bo Isomaa, Fredrik Karpe, Liming Liang, Annette Peters, Cornelia Huth, Stephen P O'Rahilly, Colin N. A. Palmer, Oluf Pedersen, Rainer Rauramaa, Jaakko Tuomilehto, Veikko Salomaa, Richard M. Watanabe, Ann-Christine Syvänen, Richard N. Bergman, Dwaipayan Bharadwaj, Erwin P. Bottinger, Yoon Shin Cho, Giriraj R. Chandak, Juliana CN Chan, Kee Seng Chia, Mark J. Daly, Shah B. Ebrahim, Claudia Langenberg, Paul Elliott, Kathleen A. Jablonski, Donna M. Lehman, Weiping Jia, Ronald C. W. Ma, Toni I. Pollin, Manjinder Sandhu, Nikhil Tandon, Philippe Froguel, Inês Barroso, Yik Ying Teo, Eleftheria Zeggini, Ruth J. F. Loos, Kerrin S. Small, Janina S. Ried, Ralph A. DeFronzo, Harald Grallert, Benjamin Glaser, Andres Metspalu, Nicholas J. Wareham, Mark Walker, Eric Banks, Christian Gieger, Erik Ingelsson, Hae Kyung Im, Thomas Illig, Paul W. Franks, Gemma Buck, Joseph Trakalo, David Buck, Inga Prokopenko, Reedik Mägi, Lars Lind, Yossi Farjoun, Katharine R. Owen, Anna L. Gloyn, Konstantin Strauch, Tiinamaija Tuomi, Jaspal Singh Kooner, Jong-Young Lee, Taesung Park, Peter Donnelly, Andrew D. Morris, Andrew T. Hattersley, Donald W. Bowden, Francis S. Collins, Gil Atzmon, John C. Chambers, Timothy D. Spector, Markku Laakso, Tim M. Strom, Graeme I. Bell, John Blangero, Ravindranath Duggirala, E. Shyong Tai, Gilean McVean, Craig L. Hanis, James G. Wilson, Mark Seielstad, Timothy M. Frayling, James B. Meigs, Nancy J. Cox, Rob Sladek, Eric S. Lander, Stacey Gabriel, Karen L. Mohlke, Thomas Meitinger, Leif Groop, Goncalo Abecasis, Laura J. Scott, Andrew P. Morris, Hyun Min Kang, David Altshuler, Noël P. Burtt, Jose C. Florez, Michael Boehnke, Mark I. McCarthy

**Affiliations:** 1 Department of Molecular Biology, Massachusetts General Hospital, Boston, Massachusetts, USA; 2 Program in Medical and Population Genetics, Broad Institute, Cambridge, Massachusetts, USA; 3 Department of Biostatistics and Center for Statistical Genetics, University of Michigan, Ann Arbor, Michigan, USA; 4 Wellcome Trust Centre for Human Genetics, Nuffield Department of Medicine, University of Oxford, Oxford, UK; 5 Harvard-MIT Division of Health Sciences and Technology, Massachusetts Institute of Technology, Cambridge, Massachusetts, USA; 6 Department of Statistics, University of Oxford, Oxford, UK; 7 Genetics of Complex Traits, University of Exeter Medical School, University of Exeter, Exeter, UK; 8 MRC Epidemiology Unit, Institute of Metabolic Science, University of Cambridge, Cambridge, UK; 9 Department of Twin Research and Genetic Epidemiology, King's College London, London, UK; 10 Oxford Centre for Diabetes, Endocrinology and Metabolism, Radcliffe Department of Medicine, University of Oxford, Oxford, UK; 11 Department of Human Genetics, Wellcome Trust Sanger Institute, Hinxton, Cambridgeshire, UK; 12 School of Computer Science, McGill University, Montreal, Quebec, Canada; 13 McGill University and Génome Québec Innovation Centre, Montreal, Quebec, Canada; 14 Human Genetics Center, The University of Texas Graduate School of Biomedical Sciences at Houston, The University of Texas Health Science Center at Houston, Houston, Texas, USA; 15 Department of Biostatistics, Boston University School of Public Health, Boston, Massachusetts, USA; 16 National Heart, Lung, and Blood Institute's Framingham Heart Study, Framingham, Massachusetts, USA; 17 Medical Genomics and Metabolic Genetics Branch, National Human Genome Research Institute, National Institutes of Health, Bethesda, Maryland, USA; 18 Department of Biostatistics, Harvard School of Public Health, Boston, Massachusetts, USA; 19 Chronic Disease Epidemiology, Swiss Tropical and Public Health Institute, University of Basel, Basel, Switzerland; 20 Institute of Genetic Epidemiology, Helmholtz Zentrum München, German Research Center for Environmental Health, Neuherberg, Germany; 21 Department of Medicine I, University Hospital Grosshadern, Ludwig-Maximilians-Universität, Munich, Germany; 22 Chair of Genetic Epidemiology, IBE, Faculty of Medicine, LMU Munich, Germany; 23 DZHK (German Centre for Cardiovascular Research), partner site Munich Heart Alliance, Munich, Germany; 24 The Novo Nordisk Foundation Center for Basic Metabolic Research, Faculty of Health and Medical Sciences, University of Copenhagen, Copenhagen, Denmark; 25 Department of Medicine, Section of Genetic Medicine, The University of Chicago, Chicago, Illinois, USA; 26 Department of Statistics, Seoul National University, Seoul, Republic of Korea; 27 Human Genetics Center, School of Public Health, The University of Texas Health Science Center at Houston, Houston, Texas, USA; 28 Saw Swee Hock School of Public Health, National University of Singapore, National University Health System, Singapore, Singapore; 29 Department of Epidemiology, Harvard School of Public Health, Boston, Massachusetts, USA; 30 Center for Genome Science, Korea National Institute of Health, Chungcheongbuk-do, Republic of Korea; 31 The Jackson Laboratory for Genomic Medicine, Farmington, Connecticut, USA; 32 Departments of Computational Medicine & Bioinformatics and Human Genetics, University of Michigan, Ann Arbor, Michigan, USA; 33 Department of Clinical Sciences, Lund University Diabetes Centre, Genetic and Molecular Epidemiology Unit, Lund University, Malmö, Sweden; 34 Department of Epidemiology, Colorado School of Public Health, University of Colorado, Aurora, Colorado, USA; 35 Department of Endocrinology and Metabolism, Shanghai Diabetes Institute, Shanghai Jiao Tong University Affiliated Sixth People's Hospital, Shanghai, China; 36 Singapore Eye Research Institute, Singapore National Eye Centre, Singapore, Singapore; 37 Department of Ophthalmology, Yong Loo Lin School of Medicine, National University of Singapore, National University Health System, Singapore, Singapore; 38 The Eye Academic Clinical Programme, Duke-NUS Graduate Medical School, Singapore, Singapore; 39 Interdisciplinary Program in Bioinformatics, Seoul National University, Seoul, Republic of Korea; 40 Department of Human Genetics, McGill University, Montreal, Quebec, Canada; 41 Research Institute of the McGill University Health Centre, Montreal, Quebec, Canada; 42 Department of Epidemiology and Biostatistics, Imperial College London, London, UK; 43 Department of Cardiology, Ealing Hospital NHS Trust, Southall, Middlesex, UK; 44 Departments of Medicine and Genetics, Albert Einstein College of Medicine, New York, USA; 45 Department of Systems Pharmacology and Translational Therapeutics, University of Pennsylvania—Perelman School of Medicine, Philadelphia, Pennsylvania, USA; 46 Department of Genetics, University of Pennsylvania—Perelman School of Medicine, Philadelphia, Pennsylvania, USA; 47 Department of Medicine, University of Texas Health Science Center, San Antonio, Texas, USA; 48 Research, South Texas Veterans Health Care System, San Antonio, Texas, USA; 49 Faculty of Health Sciences, Institute of Clinical Medicine, Internal Medicine, University of Eastern Finland, Kuopio, Finland; 50 Kuopio University Hospital, Kuopio, Finland; 51 Center for Genomics and Personalized Medicine Research, Wake Forest School of Medicine, Winston-Salem, North Carolina, USA; 52 Center for Diabetes Research, Wake Forest School of Medicine, Winston-Salem, North Carolina, USA; 53 Department of Biochemistry, Wake Forest School of Medicine, Winston-Salem, North Carolina, USA; 54 Centre for Research in Epidemiology and Population Health, Inserm U1018, Villejuif, France; 55 German Institute of Human Nutrition Potsdam-Rehbruecke, Nuthetal, Germany; 56 Department of Public Health and Caring Sciences, Geriatrics, Uppsala University, Uppsala, Sweden; 57 Centre for Chronic Disease Control, New Delhi, India; 58 The Charles Bronfman Institute for Personalized Medicine, The Icahn School of Medicine at Mount Sinai, New York, USA; 59 National Heart and Lung Institute, Cardiovascular Sciences, Hammersmith Campus, Imperial College London, London, UK; 60 Department of Genome Sciences, University of Washington School of Medicine, Seattle, Washington, USA; 61 Analytic and Translational Genetics Unit, Department of Medicine, Massachusetts General Hospital, Boston, Massachusetts, USA; 62 Center for Genomic Medicine, Department of Medicine, Massachusetts General Hospital, Boston, Massachusetts, USA; 63 Department of Psychiatry, Icahn Institute for Genomics and Multiscale Biology, Icahn School of Medicine at Mount Sinai, New York, USA; 64 Department of Public Health and Primary Care, University of Cambridge, Cambridge, UK; 65 Department of Medicine and Therapeutics, The Chinese University of Hong Kong, Hong Kong, China; 66 Department of Internal Medicine, Seoul National University College of Medicine, Seoul, Republic of Korea; 67 Department of Medicine, University of Pennsylvania, Philadelphia, Pennsylvania, USA; 68 NIHR Blood and Transplant Research Unit in Donor Health and Genomics, Department of Public Health and Primary Care, University of Cambridge, Cambridge, UK; 69 Department of Molecular Medicine and Biopharmaceutical Sciences, Graduate School of Convergence Science and Technology, and College of Medicine, Seoul National University, Seoul, Republic of Korea; 70 Department of Biostatistics and Epidemiology, University of Pennsylvania, Philadelphia, Pennsylvania, USA; 71 Center for Non-Communicable Diseases, Karachi, Pakistan; 72 Cardiovascular Division, Baylor College of Medicine, Houston, Texas, USA; 73 Department of Pediatrics, University of Texas Health Science Center, San Antonio, Texas, USA; 74 Department of Medicine, Yong Loo Lin School of Medicine, National University of Singapore, National University Health System, Singapore, Singapore; 75 Department of Epidemiology, Murcia Regional Health Council, IMIB-Arrixaca, Murcia, Spain; 76 CIBER Epidemiología y Salud Pública (CIBERESP), Spain; 77 Unit of Preventive Medicine and Public Health, School of Medicine, University of Murcia, Spain; 78 Cancer Research and Prevention Institute (ISPO), Florence, Italy; 79 Department of Medicine, University of Mississippi Medical Center, Jackson, Mississippi, USA; 80 South Texas Diabetes and Obesity Institute, Regional Academic Health Center, University of Texas Health Science Center at San Antonio/University of Texas Rio Grande Valley, Brownsville, Texas, USA; 81 Department of Genetics, Texas Biomedical Research Institute, San Antonio, Texas, USA; 82 Department of Internal Medicine, Section on Nephrology, Wake Forest School of Medicine, Winston-Salem, North Carolina, USA; 83 Center of Biostatistics and Bioinformatics, University of Mississippi Medical Center, Jackson, Mississippi, USA; 84 Department of Paediatrics, Yong Loo Lin School of Medicine, National University of Singapore, National University Health System, Singapore, Singapore; 85 Division of Human Genetics, Genome Institute of Singapore, A*STAR, Singapore, Singapore; 86 CNRS-UMR8199, Lille University, Lille Pasteur Institute, Lille, France; 87 Institute of Health Sciences, University of Oulu, Oulu, Finland; 88 Translational Laboratory in Genetic Medicine (TLGM), Agency for Science, Technology and Research (A*STAR), Singapore, Singapore; 89 Jackson Heart Study, University of Mississippi Medical Center, Jackson, Mississippi, USA; 90 College of Public Services, Jackson State University, Jackson, Mississippi, USA; 91 KG Jebsen Center for Diabetes Research, Department of Clinical Science, University of Bergen, Bergen, Norway; 92 Department of Pediatrics, Haukeland University Hospital, Bergen, Norway; 93 NIHR Biomedical Research Centre at Guy’s and St Thomas’ Foundation Trust, London, UK; 94 Institute of Human Genetics, Helmholtz Zentrum München, German Research Center for Environmental Health, Neuherberg, Germany; 95 Department of Clinical Sciences, Diabetes and Endocrinology, Lund University Diabetes Centre, Malmö, Sweden; 96 Institute of Clinical Diabetology, German Diabetes Center, Leibniz Center for Diabetes Research at Heinrich Heine University, Düsseldorf, Germany; 97 German Center for Diabetes Research (DZD), München-Neuherberg, Germany; 98 Institute of Regional Health Research, University of Southern Denmark, Odense, Denmark; 99 Department of Clinical Biochemistry, Vejle Hospital, Vejle, Denmark; 100 Department of Internal Medicine and Endocrinology, Vejle Hospital, Vejle, Denmark; 101 Department of Health, National Institute for Health and Welfare, Helsinki, Finland; 102 Abdominal Center: Endocrinology, University of Helsinki and Helsinki University Central Hospital, Helsinki, Finland; 103 Minerva Foundation Institute for Medical Research, Helsinki, Finland; 104 Department of Medicine, University of Helsinki and Helsinki University Central Hospital, Helsinki, Finland; 105 Division of Cardiovascular and Diabetes Medicine, Medical Research Institute, Ninewells Hospital and Medical School, Dundee, UK; 106 Estonian Genome Center, University of Tartu, Tartu, Estonia; 107 Department of Genetics, Harvard Medical School, Boston, Massachusetts, USA; 108 Division of Endocrinology, Boston Children's Hospital, Boston, Massachusetts, USA; 109 Nuffield Department of Primary Care Health Sciences, University of Oxford, Oxford, UK; 110 Folkhälsan Research Centre, Helsinki, Finland; 111 Research Programs Unit, Diabetes and Obesity, University of Helsinki, Helsinki, Finland; 112 Steno Diabetes Center, Gentofte, Denmark; 113 Research Centre for Prevention and Health, Capital Region of Denmark, Glostrup, Denmark; 114 Department of Public Health, Institute of Health Sciences, University of Copenhagen, Copenhagen, Denmark; 115 Faculty of Medicine, Aalborg University, Aalborg, Denmark; 116 Department of Primary Health Care, Vaasa Central Hospital, Vaasa, Finland; 117 Diabetes Center, Vaasa Health Care Center, Vaasa, Finland; 118 Institute of Epidemiology II, Helmholtz Zentrum München, German Research Center for Environmental Health, Neuherberg, Germany; 119 Research Unit of Molecular Epidemiology, Helmholtz Zentrum München, German Research Center for Environmental Health, Neuherberg, Germany; 120 Institute for Biometrics and Epidemiology, German Diabetes Center, Leibniz Center for Diabetes Research at Heinrich Heine University, Düsseldorf, Germany; 121 Department of Public Health, Section of General Practice, Aarhus University, Aarhus, Denmark; 122 Department of Clinical Experimental Research, Rigshospitalet, Glostrup, Denmark; 123 Department of Clinical Medicine, Faculty of Health and Medical Sciences, University of Copenhagen, Copenhagen, Denmark; 124 Department of Clinical Sciences, Hypertension and Cardiovascular Disease, Lund University, Malmö, Sweden; 125 Oxford NIHR Biomedical Research Centre, Oxford University Hospitals Trust, Oxford, UK; 126 Department of Clinical Sciences, Diabetes and Cardiovascular Disease, Genetic Epidemiology, Lund University, Malmö, Sweden; 127 Department of Nutrition, Harvard School of Public Health, Boston, Massachusetts, USA; 128 Channing Division of Network Medicine, Department of Medicine, Brigham and Women’s Hospital and Harvard Medical School, Boston, Massachusetts, USA; 129 Department of Epidemiology and Population Health, Albert Einstein College of Medicine, New York, USA; 130 Division of Endocrinology and Diabetology, Medical Faculty, Heinrich-Heine University, Düsseldorf, Germany; 131 Department of Public Health and Clinical Medicine, Umeå University, Umeå, Sweden; 132 High Throughput Genomics, Oxford Genomics Centre, Wellcome Trust Centre for Human Genetics, Nuffield Department of Medicine, University of Oxford, Oxford, UK; 133 Institute of Experimental Genetics, Helmholtz Zentrum München, German Research Center for Environmental Health, Neuherberg, Germany; 134 Center of Life and Food Sciences Weihenstephan, Technische Universität München, Freising-Weihenstephan, Germany; 135 William Harvey Research Institute, Barts and The London School of Medicine and Dentistry, Queen Mary University of London, London, UK; 136 Princess Al-Jawhara Al-Brahim Centre of Excellence in Research of Hereditary Disorders (PACER-HD), King Abdulaziz University, Jeddah, Saudi Arabia; 137 Department of Clinical Sciences, Medicine, Lund University, Malmö, Sweden; 138 Faculty of Health Sciences, University of Southern Denmark, Odense, Denmark; 139 Department of Social Services and Health Care, Jakobstad, Finland; 140 Metabolic Research Laboratories, Institute of Metabolic Science, University of Cambridge, Cambridge, UK; 141 Pat Macpherson Centre for Pharmacogenetics and Pharmacogenomics, Medical Research Institute, Ninewells Hospital and Medical School, Dundee, UK; 142 Foundation for Research in Health, Exercise and Nutrition, Kuopio Research Institute of Exercise Medicine, Kuopio, Finland; 143 Center for Vascular Prevention, Danube University Krems, Krems, Austria; 144 Diabetes Research Group, King Abdulaziz University, Jeddah, Saudi Arabia; 145 Dasman Diabetes Institute, Dasman, Kuwait; 146 National Institute for Health and Welfare, Helsinki, Finland; 147 Department of Preventive Medicine, Keck School of Medicine, University of Southern California, Los Angeles, California, USA; 148 Department of Physiology & Biophysics, Keck School of Medicine, University of Southern California, Los Angeles, California, USA; 149 Diabetes and Obesity Research Institute, Keck School of Medicine, University of Southern California, Los Angeles, California, USA; 150 Department of Medical Sciences, Molecular Medicine and Science for Life Laboratory, Uppsala University, Uppsala, Sweden; 151 Cedars-Sinai Diabetes and Obesity Research Institute, Los Angeles, California, USA; 152 Functional Genomics Unit, CSIR-Institute of Genomics & Integrative Biology (CSIR-IGIB), New Delhi, India; 153 Department of Biomedical Science, Hallym University, Chuncheon, Republic of Korea; 154 CSIR-Centre for Cellular and Molecular Biology, Hyderabad, Telangana, India; 155 Li Ka Shing Institute of Health Sciences, The Chinese University of Hong Kong, Hong Kong, China; 156 Hong Kong Institute of Diabetes and Obesity, The Chinese University of Hong Kong, Hong Kong, China; 157 MRC-PHE Centre for Environment and Health, Imperial College London, London, UK; 158 The Biostatistics Center, The George Washington University, Rockville, Maryland, USA; 159 Department of Medicine, Division of Endocrinology, Diabetes and Nutrition, and Program in Personalized and Genomic Medicine, University of Maryland School of Medicine, Baltimore, Maryland, USA; 160 Department of Endocrinology and Metabolism, All India Institute of Medical Sciences, New Delhi, India; 161 Department of Genomics of Common Disease, School of Public Health, Imperial College London, London, UK; 162 Life Sciences Institute, National University of Singapore, Singapore, Singapore; 163 Department of Statistics and Applied Probability, National University of Singapore, Singapore, Singapore; 164 Endocrinology and Metabolism Service, Hadassah-Hebrew University Medical Center, Jerusalem, Israel; 165 The Medical School, Institute of Cellular Medicine, Newcastle University, Newcastle, UK; 166 Department of Medical Sciences, Molecular Epidemiology and Science for Life Laboratory, Uppsala University, Uppsala, Sweden; 167 Hannover Unified Biobank, Hannover Medical School, Hanover, Germany; 168 Department of Human Genetics, Hannover Medical School, Hanover, Germany; 169 Department of Medical Sciences, Uppsala University, Uppsala, Sweden; 170 Data Sciences and Data Engineering, Broad Institute, Cambridge, Massachusetts, USA; 171 Finnish Institute for Molecular Medicine, University of Helsinki, Helsinki, Finland; 172 Imperial College Healthcare NHS Trust, Imperial College London, London, UK; 173 Clinical Research Centre, Centre for Molecular Medicine, Ninewells Hospital and Medical School, Dundee, UK; 174 The Usher Institute to the Population Health Sciences and Informatics, University of Edinburgh, Edinburgh, UK; 175 University of Exeter Medical School, University of Exeter, Exeter, UK; 176 Department of Natural Science, University of Haifa, Haifa, Israel; 177 Institute of Human Genetics, Technische Universität München, Munich, Germany; 178 Departments of Medicine and Human Genetics, The University of Chicago, Chicago, Illinois, USA; 179 Cardiovascular & Metabolic Disorders Program, Duke-NUS Medical School Singapore, Singapore, Singapore; 180 Li Ka Shing Centre for Health Information and Discovery, University of Oxford, Oxford, UK; 181 Department of Physiology and Biophysics, University of Mississippi Medical Center, Jackson, Mississippi, USA; 182 Department of Laboratory Medicine & Institute for Human Genetics, University of California, San Francisco, San Francisco, California, USA; 183 Blood Systems Research Institute, San Francisco, California, USA; 184 General Medicine Division, Massachusetts General Hospital and Department of Medicine, Harvard Medical School, Boston, Massachusetts, USA; 185 Division of Endocrinology and Metabolism, Department of Medicine, McGill University, Montreal, Quebec, Canada; 186 Broad Institute of MIT and Harvard, Cambridge, Massachusetts, USA; 187 Department of Genetics, University of North Carolina, Chapel Hill, North Carolina, USA; 188 Department of Biostatistics, University of Liverpool, Liverpool, UK; 189 Department of Medicine, Harvard Medical School, Boston, Massachusetts, USA; 190 Diabetes Research Center (Diabetes Unit), Department of Medicine, Massachusetts General Hospital, Boston, Massachusetts, USA; 191 Department of Biology, Massachusetts Institute of Technology, Cambridge, Massachusetts, USA

**Keywords:** DNA sequencing, Type 2 diabetes, Genome-wide association studies

## Abstract

To investigate the genetic basis of type 2 diabetes (T2D) to high resolution, the GoT2D and T2D-GENES consortia catalogued variation from whole-genome sequencing of 2,657 European individuals and exome sequencing of 12,940 individuals of multiple ancestries. Over 27M SNPs, indels, and structural variants were identified, including 99% of low-frequency (minor allele frequency [MAF] 0.1–5%) non-coding variants in the whole-genome sequenced individuals and 99.7% of low-frequency coding variants in the whole-exome sequenced individuals. Each variant was tested for association with T2D in the sequenced individuals, and, to increase power, most were tested in larger numbers of individuals (>80% of low-frequency coding variants in ~82 K Europeans via the exome chip, and ~90% of low-frequency non-coding variants in ~44 K Europeans via genotype imputation). The variants, genotypes, and association statistics from these analyses provide the largest reference to date of human genetic information relevant to T2D, for use in activities such as T2D-focused genotype imputation, functional characterization of variants or genes, and other novel analyses to detect associations between sequence variation and T2D.

## Background & Summary

Genome wide association studies (GWAS) have provided a valuable but incomplete window into the genetic basis of type 2 diabetes (T2D)^1^. Common (minor allele frequency [MAF]>5%) variants at over 100 loci have been robustly associated with disease risk, but most have not yet been translated to causal variants, effector transcripts, or disease mechanisms^2^. Because common variants from GWAS have modest effect sizes, and because those previously published explain in aggregate only 10–15% of the genetic basis of T2D^3^, it has been hypothesized that variants unexplored by GWAS might have a greater impact on efforts to understand or treat T2D^4,5^.

**Figure 1:**
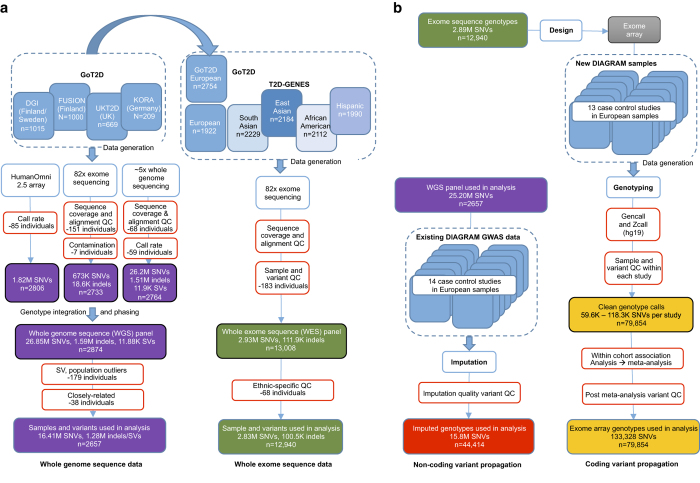
Overview of data and analysis generation. Shown is a flowchart for variant calling, quality control, and propagation of variants in both the WGS panel and WES panel. (**a**) Variant calling and quality control in the WGS and WES panels. Individuals were characterized with one or more sequencing and genotyping technologies, and then individuals and variants were excluded based on quality control metrics. The final WGS panel consists of data from 2,857 individuals and 28.5M variants, while the final WES panel consists of data from 13,008 individuals and 3.04M variants. (**b**) Assessing variants in larger sample sizes. Non-coding variants from the WGS panel were studied via statistical imputation in 44,414 additional individuals from cohorts within the DIAGRAM consortium. Coding variants from the WES panel were genotyped on the exome array in 79,854 additional individuals. Modified from Extended Data Fig. 1 of Fuchsberger *et al.*^8^.

To produce a more complete catalogue of rare, low-frequency, and common variants, the GoT2D and T2D-GENES consortia analysed whole-exome and genome sequence data in up to 12,940 individuals (6,504 T2D cases and 6,436 controls; Fig. 1a, Tables 1 and 2)^3^. First, to interrogate lower-frequency (MAF>0.5%) variation genome-wide, 2,657 Northern and Central European individuals were selected by GoT2D (1,326 cases, 1,331 controls) and characterized with a combination of low-pass (~5x) whole-genome sequencing, deep (~82x) whole-exome sequencing, and high-density (2.5M) SNP genotyping. Genetic variants from these assays were incorporated into a phased integrated panel (*WGS panel*), capturing an estimated 99% of variants, genome-wide, present in more than 0.5% of individuals (Table 3). Second, additional individuals from 10 cohorts spanning five ethnicities (European, Hispanic, South Asian, East Asian, and African American) were characterized by deep (~82x) whole-exome sequencing by T2D-GENES. The resultant T2D-GENES exome sequence data were combined with the GoT2D exome sequence data to produce a second panel of variation (*WES panel*), capturing an estimated 99.7% of coding variants present in more than 0.5% of the combined 12,940 individuals (Tables 4 and 5).

Each variant was tested for association with T2D under an additive genetic model. To increase power, variants were then assessed in larger sample sizes via one of two means (Fig. 1b). Coding variants were analysed in 79,854 additional individuals (28,305 T2D cases, 51,549 controls) via the Illumina Exome Array, which captures 81.6% of European MAF>0.5% coding variants in the WES panel. Non-coding variants (and coding variants absent from the Exome Array) were analysed in up to 44,414 additional individuals (11,645 cases, 32,769 controls) via statistical genotype imputation; after quality control, this analysis included 89% of variants observed in three or more individuals in the WGS panel. Each variant was tested for association with T2D in the additional individuals, under an additive genetic model, and association statistics were then combined with those from the sequence data via meta-analysis.

Collectively, these analyses suggest a limited role for low-frequency variation in the genetic basis of T2D^3^. However, they also demonstrate an ability to identify novel hypotheses about the effects of gene inactivation^6^, a resource of coding variants for calibrating cellular assays^7^, and a catalogue of noncoding variants for use in statistical or functional fine mapping of GWAS signals^3^. The WGS panel also provides a novel resource for genotype imputation^8^, with increased resolution for T2D-specific variants relative to the 1000 Genomes (1000G) reference panel, as well as a means to calibrate simulation-based models of population history^9^ or disease genetic architecture^10^.

## Methods

These methods are a modified version of the descriptions contained in Fuchsberger *et al.*^3^.

### Ethics statement

All human research was approved by the relevant institutional review boards and conducted according to the Declaration of Helsinki. All participants provided written informed consent.

### WGS GoT2D integrated panel generation

#### Ascertainment of individuals

Individuals were sampled from four studies: the Finland-United States Investigation of NIDDM Genetics (FUSION) Study (493 cases, 486 controls), KORA (101 cases, 104 controls), the UKT2D Consortium (322 cases, 322 controls), and the Malmö-Botnia Study (410 cases, 419 controls). All individuals were of Northern or Central European ancestry. Cases were preferentially lean, had (relatively) early onset T2D, or had a familial history of T2D; controls by comparison were preferentially overweight or had low fasting glucose levels^11^. To decrease the likelihood of selecting T2D cases who in fact had type 1 diabetes (T1D) or monogenic forms of diabetes (such as Maturity Onset Diabetes of the Young), cases with an age of diagnosis below 35, testing positive for GAD antibodies, or with a first-degree relative known to have T1D were not included. Statistics of the 2,657 individuals ultimately included in the association analysis are provided in Table 1.

Many of these individuals were measured for cardiometabolic phenotypes other than T2D, including glucose and insulin, anthropometrics, lipids, and blood pressure (Table 6).

#### DNA sample preparation

De-identified DNA samples were sent to the Broad Institute in Cambridge, MA, USA (Malmö-Botnia and FUSION), the Wellcome Trust Centre for Human Genetics in Oxford, UK (UKT2D), or the Helmholtz Zentrum München in Germany (KORA). DNA quantity was measured by Picogreen (all samples) to ensure sufficient total DNA and minimum concentrations for downstream experiments. Samples (Malmö-Botnia, FUSION, UKT2D) were then genotyped on a Sequenom iPLEX assay for a set of 24 SNPs (one X chromosome and 23 autosomal SNPs), with only samples with high-quality genotypes advanced for subsequent sequencing or genotyping.

#### Exome sequencing

Genomic DNA was sheared, end repaired, ligated with barcoded Illumina sequencing adapters, amplified, size selected, and subjected to in-solution hybrid capture using the Agilent SureSelect Human All Exon v2.0 (Malmö-Botnia, FUSION, UK2T2D) or v3.0 (KORA) bait set (Agilent Technologies, USA). Resulting Illumina exome sequencing libraries were qPCR quantified, pooled, and sequenced with 76-bp paired-end reads using Illumina GAII or HiSeq 2,000 sequencers to ~82-fold mean coverage.

#### Genome sequencing

Whole-genome Illumina sequencing library construction was performed as described for exome sequencing above, except that genomic DNA was sheared to a larger target size and hybrid capture was not performed. The resulting libraries were size selected to contain fragment insert sizes of 380 bp±20% (Malmö-Botnia, FUSION, KORA) and 420 bp±25% (UKT2D) using gel electrophoresis or the SAGE Pippin Prep (Sage Science, USA). Libraries were qPCR quantified, pooled, and sequenced with 101-bp paired-end reads using Illumina GAII or HiSeq 2,000 sequencers to ∼5-fold mean coverage.

#### HumanOmni2.5 array genotyping and quality control (QC)

SNP array genotyping was performed by the Broad Genetic Analysis Platform. DNA samples were placed on 96-well plates and assayed using the Illumina HumanOmni2.5-4v1_B SNP array. Genotypes were then called using Illumina GenomeStudio v2010.3 with default clusters. SNPs with GenTrain score <0.6, cluster separation score <0.4, or call rate <97% were considered technical failures at the genotyping laboratory and excluded from further analysis. Next, 85 individuals with a genotype call rate below 98%, low genetic fingerprint (24-marker panel) concordance, or estimated gender discordance were excluded from further analysis. Finally, SNPs monomorphic across all individuals, failed by the 1000G Omni 2.5 QC filter, or with Hardy-Weinberg equilibrium *P*<10^−6^ were excluded from analysis.

#### Processing, quality control, and variant calling of sequence data

Sequence data were processed and aligned to the human genome (build hg19) using the Picard (http://broadinstitute.github.io/picard/), BWA^12^, and GATK^13,14^ software packages, following best-practice pipelines.

Sequencing coverage of each individual was computed based on the fraction of target bases with >20 reads aligned (exome sequencing) or average number of reads aligned across all bases genome-wide (genome sequencing). Based on these metrics, we excluded from further analysis exome sequence data (from 151 individuals) with coverage ≤20x in >20% of the target bases and genome sequence data (from 68 individuals) with average coverage ≤5x.

Possible DNA contamination of sequence data was assessed using verifyBamID^15^, either by direct comparison of sequence and HumanOmni2.5 array genotypes (where available) or by indirect estimates of contamination based on HumanOmni2.5 array allele frequencies. DNA samples with estimated contamination >2% using either method were excluded from further analysis (data from 7 individuals in the exome sequencing dataset and 59 individuals in the genome sequencing dataset). Uncontaminated DNA sample swaps were also detected via comparison of sequence and array data and corrected prior to variant calling.

To identify single nucleotide variants (SNVs) from the whole-genome sequence data, we used two independent SNV calling pipelines: GotCloud^16^ and the GATK UnifiedGenotyper^14^. We merged unfiltered SNV calls across the two call sets and then processed the merged site list through the SVM and VQSR filtering algorithms implemented by those pipelines. SNVs that failed both filtering algorithms were excluded from further analysis. To identify SNVs from the whole-exome sequence data, we used the GATK UnifiedGenotyper best-practices pipeline^14^.

To identify short insertions and deletions (indels) from the whole-genome sequence data, we called variants using the GATK UnifiedGenotyper best-practices pipeline. Because indels are known to have high false positive rates^17^, we applied more stringent criteria for indel QC than for SNV QC, excluding indels that failed either the SVM or VQSR filtering algorithms. To identify indels from the whole-exome sequence data, we used the GATK UnifiedGenotyper best-practices pipeline^14^.

To identify structural variants (SVs, or >100-bp deletions) from the whole-genome sequence data, we used GenomeSTRiP^18^. To increase sensitivity after initial discovery of SVs, we merged the discovered sites with deletions identified in 1,092 sequenced individuals from the 1000G Project^17^ and then genotyped the merged site lists across the whole-genome sequenced individuals. After applying the default filtering implemented in GenomeSTRiP, pass-filtered sites variable in any of the individuals were identified as candidate variant sites. Among these candidate sites, we excluded variants in known immunoglobin loci to reduce the impact of possible cell-line artifacts. We did not call SVs from the whole-exome sequence data.

#### Integrated panel generation

We merged variants discovered from the three experimental platforms into one site list. For individuals who had data from each of the three platforms, we then calculated genotype likelihoods across all sites separately by platform: for the whole-genome sequence data, we used GotCloud; for the exome sequence data, we used the GATK UnifiedGenotyper; and for the HumanOmni2.5 data, we converted hard genotype calls into genotype likelihoods assuming a genotype error rate of 10^−6^. If a site was not assayed by one of the three platforms, it was ignored in likelihood calculation for that platform.

We then calculated combined genotype likelihoods as the product of the genome, exome, and HumanOmni2.5 likelihoods, assuming independence across platforms. Following a strategy originally developed for the 1000G Phase 1 project^17^, we then phased the integrated likelihoods using Beagle^19^ (with 10,000 SNVs per chunk and 1,000 overlapping SNVs between consecutive chunks) and refined phased genotypes using Thunder^20^ as implemented in GotCloud (with 400 states).

Using the genotypes from the integrated panel, we performed principal component analysis (PCA) separately for each of the three variant types (SNVs, indels, SVs), using EPACTS on an LD-pruned (*r*^2^<0.20) set of MAF>0.01 autosomal variants (with variants in large high-LD regions^21,22^ or with Hardy-Weinberg *P*<10^−6^ removed). Inspecting the first ten PCs for each variant type, we identified 43 outlier individuals based on PCs from SNVs and indels and 136 additional outliers based on PCs from SVs; these 179 individuals were excluded from further analysis. Additionally, 38 individuals with close relationships with other study individuals (estimated genome-wide identity-by-descent proportion of alleles shared >0.20) were excluded from further analysis.

The final WGS panel contains genotypes from 2,874 individuals at 26.85M SNVs, 1.59M indels, and 11.88 K SVs. The final analysis set includes genotypes from 2,657 individuals at 26.20M SNVs, 1.50M indels, and 8.88K SVs SVs (Table 3).

### WES (GoT2D+T2D-GENES Multiethnic) panel generation

#### Ascertainment of individuals

In addition to the individuals within the WGS panel, additional individuals, 10,242 of which were included in the final analysis, were chosen for whole-exome sequencing from 10 studies: the Jackson Heart Study (500 African-American cases, 526 matched controls), the Wake Forest School of Medicine Study (518 African-American cases, 530 matched controls), the Korea Association Research Project (526 East-Asian cases, 561 matched controls), the Singapore Diabetes Cohort Study and Singapore Prospective Study Program (486 East-Asian cases, 592 matched controls), Ashkenazi (506 European cases, 355 matched controls), the Metabolic Syndrome in Men (METSIM) Study (484 European cases, 498 matched controls), the San Antonio Mexican American Family Studies (272 Hispanic cases, 218 matched controls), the Starr County Texas study (749 Hispanic cases, 704 matched controls), the London Life Sciences Population (LOLIPOP) Study (531 South-Asian cases, 538 matched controls), and the Singapore Indian Eye Study (563 South-Asian cases, 585 matched controls). Potential T1D or MODY cases were excluded via similar approaches as for the whole-genome sequencing experiment. Statistics of the individuals ultimately included in the association analysis are provided in Table 2.

As for the WGS panel, many individuals were measured for additional cardiometabolic phenotypes (Table 6).

#### Exome sequencing

DNA samples were obtained and sequenced in the same manner as described for the exome sequencing component of the WGS panel.

#### Processing, QC, and variant calling

As for the exome sequence data within the WGS panel, sequence data for the WES panel were processed and aligned to the human genome (build hg19) using the Picard, BWA^12^, and GATK^13,14^ software packages and best-practice pipelines. Genotype likelihoods were computed controlling for contamination. Hard calls (the GATK-called genotypes but set as missing at a genotype quality [GQ] <20 threshold^14^) and dosages (the expected value of the genotype, defined as *Pr*(RX|data)+2*Pr*(XX|data), where R is the reference and X the alternative allele) were computed for each individual at each variant site. Hard calls were used only for quality control, while dosages were used in downstream association analyses. Multi-allelic SNVs and indels were dichotomized by collapsing alternate alleles into one category.

Individuals were excluded from analysis if they were outliers on one of multiple metrics: poor array genotype concordance (where available), high number of variant alleles or singletons, high or low allele balance (average proportion of non-reference alleles at heterozygous sites), or excess mean heterozygosity or ratio of heterozygous to homozygous genotypes. Within this reduced set of individuals, we then further excluded variants based on hard call rate (<90% in any cohort), deviation from Hardy-Weinberg equilibrium (*P*<10^−6^ in any ancestry group), or differential call rate between T2D cases and controls (*P*<10^−4^ in any ancestry group).

The final WES panel contains genotypes for 13,008 individuals at 2.93M SNVs and 111.9 K indels. The set ultimately included in coding variant association analysis (after removal of individuals with close relatives or of uncertain ancestry) contains genotypes for 12,940 individuals at 2.89M SNVs and 110.2 K indels (Tables 4 and 5).

### Assaying variants in larger sample sizes

#### Imputation from the WGS panel

We carried out genotype imputation, using existing SNP array data, from the WGS panel into 44,414 individuals (11,645 T2D cases and 32,769 controls) from 13 studies participating in the DIAGRAM consortium. Each study performed quality control independently. A more detailed description of the analyzed individuals is available elsewhere^3^.

#### Exome array genotyping from the WES panel

We considered 28,305 T2D cases and 51,549 controls from 13 studies of European ancestry, each genotyped with the Illumina exome array. Studies independently called genotypes using the Illumina GenCall algorithm (http://www.illumina.com/Documents/products/technotes/technote_gencall_data_analysis_software.pdf ) or Birdseed^23^. Individuals were excluded if they had a low call rate (<99%), excess heterozygosity, high singleton counts, evidence of non-European ancestry, discrepancy between recorded and genotyped sex, or discordance with prior SNP array or genotyping platform fingerprint data (where available). Variants were excluded if they had a low call rate (<99%), deviation from Hardy-Weinberg equilibrium (*P*<10^−6^), GenTrain score <0.6, cluster separation score <0.4, or a suspect intensity plot based on manual inspection. After quality control, missing genotypes were re-called using zCall^24^, and additional quality control was performed to exclude poorly genotyped individuals (call rate <99% or excess heterozygosity) or variants (call rate <99%). A more detailed description of the analyzed individuals is available elsewhere^3^.

### Association analysis

#### WGS panel single variant analysis

For each variant in the WGS panel, we tested for association between genotype and T2D in the 2,657 sequenced individuals. We used a logistic regression framework (assuming an additive genetic model) with the Firth bias-corrected likelihood ratio test^25,26^ to test for significance. Tests were adjusted for sex, the first two PCs computed based on genotypes from the HumanOmni2.5M array, and an indicator function for observed temporal stratification based on sequencing date and center.

#### Analysis of imputed datasets

In each of the thirteen studies within which variants from the WGS panel were imputed, SNVs with minor allele count (MAC)≥1 were tested for T2D association under an additive genetic model. Association tests were adjusted for study-specific covariates and performed using either the Firth, likelihood ratio, or score tests as implemented in EPACTS (https://genome.sph.umich.edu/wiki/EPACTS) or SNPTEST^27^. Residual population stratification for each study was accounted for using genomic control^28^. Association statistics were then combined across studies, using a fixed-effects sample-size weighted meta-analysis as implemented in METAL^29^.

#### WES panel single variant analysis

For each variant in the WES panel, we tested for association between genotype and T2D in the 12,940 sequenced individuals. We computed separate association statistics for each ancestry group using EMMAX^30^. Additionally, we performed association tests using the Wald statistic, adjusting for ethnic-specific principal components after exclusion of related individuals. For each test, we calculated genomic control inflation factors and corrected association summary statistics (*P*-values and standard errors) to account for residual population structure.

We subsequently performed a fixed-effects meta-analysis of ancestry-specific association summary statistics for each variant using (i) a sample-size weighting of *P*-values from the EMMAX analysis and (ii) an inverse-variance weighting of effect size estimates from the Wald analysis. For the final results, *P*-values were taken from the EMMAX analysis, and effect size estimates from the Wald analysis.

#### Analysis of exome array datasets

In each study within which exome array genotyping was applied, variants were tested for association with T2D via both the EMMAX and Wald tests. For the Wald test, related individuals were excluded and statistics were adjusted for study-specific principal components. For each study, *P*-values and standard errors were corrected based on the calculated genomic control inflation factor.

Variants were then combined via a fixed-effects meta-analysis. EMMAX *P*-values were combined via a sample-size weighted analysis, and Wald effect sizes were combined via an inverse-variant weighted analysis. For the final results, *P*-values were taken from the EMMAX analysis, and effect sizes were estimated from the Wald analysis.

#### Gene-level analysis

We first generated four variant lists (‘masks’) based on functional annotations and observed allele frequencies. Annotations were computed based on transcripts in ENSEMBL 66 (GRCh37.66) using CHAoS v0.6.3, SnpEFF v3.1^31^, and VEP v2.7^32^. We then identified variants predicted by at least one of the three algorithms in at least one mapped transcript to be protein-truncating ('for example, nonsense, frameshift, essential splice site), denoted PTVs, or other protein-altering (for example, missense, in-frame indel, non-essential splice site), denoted missense. We additionally used a previously described procedure^33^ to identify subsets of missense variants bioinformatically predicted to be deleterious: those annotated as damaging by each of Polyphen2-HumDiv, PolyPhen2-HumVar, LRT, Mutation Taster, and SIFT were considered to meet ‘strict’ criteria, while those annotated as damaging by one of these algorithms was considered to meet ‘broad’ criteria. We then calculated the MAF of each variant based on the highest frequency across each of the five ancestry groups. We finally combined these annotations to produce four masks: the PTV-only mask included PTVs, the PTV+NS_strict_ mask included variants in the PTV-only mask as well as those meeting ‘strict’ criteria for deleteriousness, the PTV+NS_broad_ mask included variants in the PTV-only mask as well as those with MAF<1% meeting ‘broad’ criteria for deleteriousness, and the PTV+missense mask included variants in the PTV+NS_broad_ mask as well as those with MAF<1% annotated as missense.

We performed gene-level analysis using the MetaSKAT software package (v0.32)^34^, employing the SKAT v0.93 library to perform a SKAT-O^35^ analysis within each ancestry group as well as across all ancestry groups via meta-analysis. Within each ancestry group, we assumed homogenous allele frequencies and genetic affects and adjusted for ethnic-specific axes of genetic variation after exclusion of 96 related individuals. For the meta-analysis, we used the MetaSKAT option to analyze genotype-level data, allowing for heterogeneity of allele frequencies and genetic effects between ancestry groups. All analyses were completed using the recommended *ρ* vector for SKAT-O: (0, 0.12, 0.22, 0.32, 0.52, 0.5, 1).

### Code availability

All analyses were performed using publically available software packages, using versions and parameters as described above.

## Data Records

Genotypes and phenotypes from the WGS and WES panels are available at the European Genome-phenome Archive (EGA, Data Citation 1 and Data Citation 2) and the database of Genotypes and Phenotypes (dbGAP, Data Citation 3 to Data Citation 12).

The data in EGA are covered under a single data use agreement, which complies with all of the cohort-specific data use restrictions. While this does limit data access according to the criteria of the most restrictive cohort, it is the only mechanism through which the entire WES and WGS panels are available to investigators. Additionally, the EGA contains data from one cohort that could not be released to a US-based repository. To download either the WGS or WES panel from the EGA, investigators must obtain approval from a data access committee (DAC, t2dgenes-got2d-dac@broadinstitute.org) to analyze data from all cohorts included in the study. The requester will receive an application packet that includes a project proposal document and a Data Transfer Agreement (DTA). The requester must then provide to the DAC a short description of their study, the proposed use of the data, an approval from the Institution's IRB, and a signed DTA. Assuming IRB approval and an executed DTA, the process for obtaining final approval from the DAC takes 4–6 weeks. Once approved, investigators can download either a single VCF file with genotypes from all individuals in the WGS panel (Data Citation 1) or a single VCF file with genotypes from all individuals in the WES panel (Data Citation 2).

If investigators cannot obtain approval to analyze all cohorts in the WES panel (e.g., commercial uses) they can download cohort-specific data from dbGAP. Each cohort in dbGAP is subject to distinct data use restrictions, and investigators can obtain separate VCF files, as well as the raw sequence reads, for each of the cohorts.

The WGS and WES panels are accompanied by exclusion lists of variants and individuals (Data Citation 1 and Data Citation 2). The WGS VCF file contains data from the full set of 2,874 individuals and 28.45M variants that passed QC, with additional lists provided containing the 2,657 individuals and 17.69M variants included in association analysis. The WES VCF file contains the full set of 13,008 individuals that passed QC, with additional lists provided containing the 3.04M variants that passed QC (the VCF includes a small number of variants that failed QC) and the 12,940 samples and 2.93M variants included in association analysis. Additionally, the WES dataset includes lists of variants included in gene-level analysis for each of the four analyzed masks.

Five datasets of association statistics are also available for download. Association statistics for variants in the WGS panel are available from the whole-genome sequenced individuals or from those with imputed genotypes (Data Citation 1). Association statistics in the WES panel are available from the whole-exome sequenced individuals or from those genotyped on the exome array (Data Citation 2). Additionally, gene-level association statistics from the whole-exome sequenced individuals are available for each of the four variant masks (Data Citation 2).

A description of the datasets is available in Table 7. All association statistics are also available for browsing and searching via the public Type 2 Diabetes Knowledge Portal at www.type2diabetesgenetics.org. Through the portal, users can construct queries to find variants satisfying specified annotations and association thresholds, both across the WES and WGS analyses as well as other GWAS datasets. Users can also dynamically construct a set of variants within a gene and obtain a *P*-value from aggregate association analysis within the WES individuals.

## Technical Validation

### Evaluation of variants in the WGS panel

**Figure 2:**
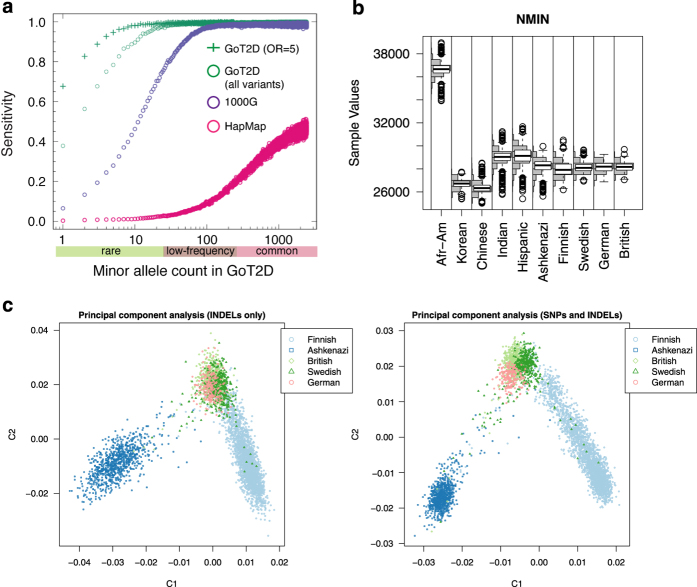
Summary of key quality control metrics for WGS and WES panels. We computed several metrics to verify the sequencing accuracy of the study individuals. (**a**) Estimates of sensitivity of WGS panel. Shown is the fraction of variants, as a function of minor allele count in the WGS sequenced individuals, estimated as included in the WGS panel. Green circles show the total fraction of variants; green crosses show the fraction of variants for hypothetical variants with a T2D odds ratio of 5 (because T2D cases are overrepresented in our sample, the sensitivity to detect risk variants is increased). For comparison, shown are the fraction of variants that are included in the 1000G Phase 1 dataset (blue circles) or HapMap panel (red circles). (**b**) Distribution of minor alleles carried by individuals in the WES panel. For different populations within the WES panel, the distribution of minor alleles carried is plotted across all individuals. A normal distribution indicates a lack of systematic sequencing artefacts for any one individual, at least according to this metric. Afr-Am: African American. (**c**) Comparison of principal components computed from SNPs and indels versus indels alone. We calculated principal components for the European individuals in the WES panel using all variants in the panel and then again using only indels. Adapted from Supplementary Tables 5 and 6, and Fig. 1a, in Fuchsberger *et al.*^8^.

We evaluated the variant sensitivity (fraction of true variant sites detected) of the WGS panel, based on the 2,538 individuals with data from all three experimental platforms (low-pass whole-genome sequencing, whole-exome sequencing, and HumanOmni2.5M array genotyping). To assess the sensitivity of low-pass whole-genome sequencing alone, we computed the fraction of variants detected from whole-exome sequencing that were also detected by low-pass whole-genome sequencing. Sensitivity estimates were 99.8, 99.0, and 48.2% for common (MAF>5%), low-frequency (0.5%<MAF<5%), and rare (MAF<0.5%) SNVs, respectively, and >99.9, 93.8, and 17.9% for common, low-frequency, and rare short indels, respectively. We also assessed the coding SNV sensitivity of low-pass whole-genome sequence data combined with exome sequence data, based on the proportion of HumanOmni2.5 SNVs detected by either sequencing platform. Because HumanOmni2.5 SNVs are enriched for common variants, we calculated an averaged sensitivity at each allele count, weighted by the number of exome-detected variants given the allele count. Sensitivity estimates were 99.9, 99.7, and 83.9% for common, low-frequency, and rare variants, respectively. These sensitivity estimates provide lower bounds on the sensitivity of the full WGS panel, which combines HumanOmni2.5 SNP array data as well as the two types of sequence data (Fig. 2a).

We further evaluated the genotype accuracy of the WGS panel for each of the three classes of variant (SNVs, indels, and SVs). Across chromosome 20, concordance of low-pass whole-genome-sequence-based SNV genotypes with exome-sequence-based genotypes was 99.86%, with homozygous reference, heterozygous, and homozygous non-reference concordances of 99.97, 98.34, and 99.72%, respectively. Concordance between exome-sequence-based SNV genotypes and HumanOmni2.5 genotypes was 99.4%, with homozygous reference, heterozygous, and homozygous non-reference concordances of 99.97, 99.69, and 99.88%, respectively. For indels genotyped with both low-pass whole-genome-sequence data and exome-sequence data, concordance was 99.4%, with homozygous reference, heterozygous, and homozygous non-reference concordances of 99.8, 95.8, and 98.6%, respectively.

To evaluate the genotype accuracy of SVs detected from the low-pass whole-genome sequence data, we took advantage of the 181 individuals in our study who were previously included in the WTCCC array-CGH based structural variant detection experiment^36^. Taking the WTCCC data as a gold standard, we estimated genotype accuracy across 1,047 overlapping SVs (with reciprocal overlap >0.8) genome-wide. The overall genotype concordance was 99.8%, with homozygous reference, heterozygous, and homozygous non-reference concordances of 99.9, 99.6, and 99.7%, respectively.

### Evaluation of variants in the WES panel

We assessed the overall sequencing quality of individuals in the WES panel by computing distributions of global statistics, stratified by reported ancestry (Fig. 2b). After quality control, the number of non-reference variants, mean heterozygosity, and average allele balance (fraction of non-reference reads at heterozygous sites) per individual approximately matched a Gaussian distribution within each ancestry. Concordance between genotypes from exome-sequence data and those from independent SNP arrays was above 99% for the vast majority of individuals, with non-reference concordance above 99.5% for individuals genotyped on the (highest-quality) OMNI array.

We also assessed bulk properties of indels within the WES panel. The length distribution of indels showed an excess of variants with lengths a multiple of three, as expected. Additionally, principal components computed from indels alone closely matched those computed from SNVs and indels together (Fig. 2c).

### Evaluation of imputation from the WGS panel

**Figure 3:**
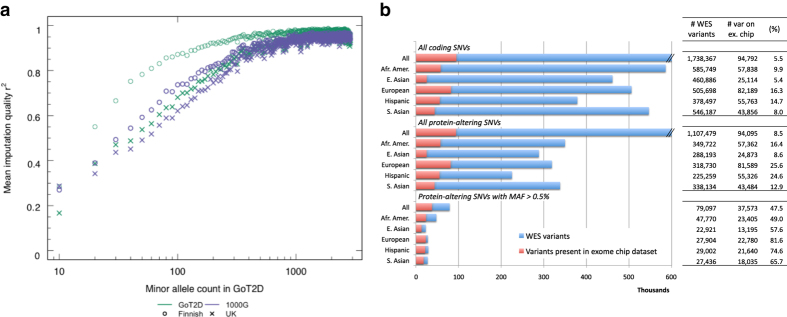
Completeness of additional variant genotyping. We calculated the fraction of variants in the WGS and WES panel that were captured via either imputation or exome array genotyping, respectively. (**a**) The mean imputation quality of variants in the WGS panel, as a function of their allele count in the WGS panel. Green circles show imputation quality in Finnish individuals, while green crosses show imputation quality in British individuals. For comparison, blue circles and crosses show imputation quality using the 1000G Phase 1 dataset as a reference panel (instead of the WGS panel). (**b**) The number of coding variants in the WES panel present on the exome array. Variants are stratified by annotation and frequency, and sensitivity calculations are shown for variants in each ancestry group as well as overall. Panel (**b**) is reproduced from Supplementary Fig. 17 in Fuchsberger *et al.*^8^.

We computed the mean imputation quality as measured by the average squared correlation between imputed genotypes and actual genotypes from leave-one-out cross-validation analysis. For variants of allele count ≈100 or above in the WGS panel (corresponding to a frequency of 1.8%), average r^2^ values were in excess of 0.8 for Finnish individuals and in excess of 0.6 for British individuals (Fig. 3a).

### Evaluation of exome array sensitivity

We assessed the overlap of variants present on the exome array with those observed in the WES panel. As the exome array primarily contains SNVs that are predicted to be protein altering, we focused on nonsense, essential splice site, and missense variants; only variants passing QC in both sequence and array data were included in the assessment. The fraction of variants in the WES panel on the exome array was highest for Europeans, at 81.6%, and lowest in African-Americans, at 49.0% (Fig. 3b).

### Evaluation of association tests

**Figure 4:**
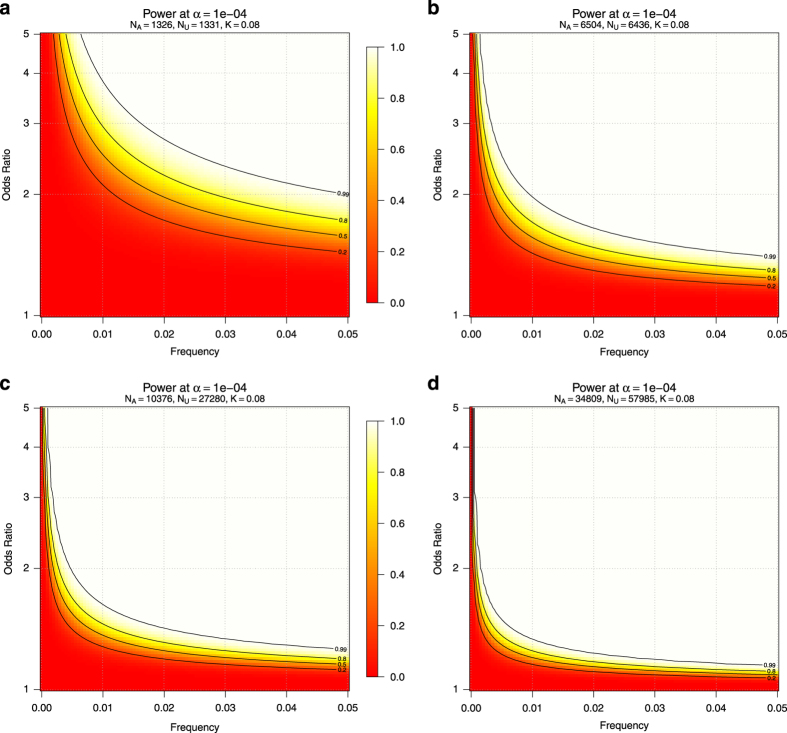
Power of single variant analysis in the WGS panel, WES panel, imputation, and exome array analyses. Shown is the power to detect an association with variants of varying population frequencies and T2D odds ratios, at a relatively lenient significance level of α=10^−4^. Such a significance level would be insufficient to establish an association due to the burden of multiple testing, but lack of association at this significance level can place bounds on the maximum effect a variant has in the population. (**a**) Power for a variant of constant frequency and effect across all populations in the WGS panel. (**b**) Power for a variant of constant frequency and effect across all populations in the WES panel. (**c**) Power for a variant imputed from the WGS panel with imputation accuracy r^2^=0.8. (**d**) Power for a variant in both the WES panel and on the exome array. N_A_: number of affecteds (cases); N_U_: number of unaffecteds (controls); K: presumed prevalence of T2D in the population.

We used the genetic power calculator (http://zzz.bwh.harvard.edu/gpc/) to estimate power to detect T2D association for each of the single variant analyses. All calculations assumed a T2D prevalence of 8%. Figure 4 shows power estimates under optimistic scenarios, in which a variant is present in the WGS panel (Fig. 4a), observed at equal frequency in all ancestries in the WES panel (Fig. 4b), imputed with high (r^2^=0.8) accuracy (Fig. 4c), or included on the exome array (Fig. 4d). We also computed power for less optimistic scenarios^3^; if, for example, a MAF 1% variant is present in only one ancestry, it must have an odds ratio of ~3.5 to achieve significance of *P*=10^−4^, rather than an odds ratio of ~1.8 were it to be present in all ancestries.

For gene-based tests in the WES panel, we used a simulated haplotype data set (http://cran.r-project.org/web/packages/SKAT/vignettes/SKAT.pdf) and estimated power as a function of (i) the phenotypic variance, under a liability scale, explained by additive genetic effects and (ii) the percentage of variants that were causal (50% or 100%). As for single-variant power calculations, we considered variants of constant frequency across all five ancestry groups, as well as variants specific to one ancestry group. These calculations suggested^3^ that, even under optimistic scenarios, genes must explain >1% of genetic variance in order to achieve a moderately significant (*P*<10^−3^) association in the WES panel.

To ensure that association statistics were well calibrated, we computed quantile-quantile (QQ) plots comparing observed statistics to those expected under the null distribution. The vast majority of statistics matched the expected distribution (suggesting good calibration of the association tests) with a deviation from the null for common variant associations from the WGS panel, imputed genotypes, and exome array genotypes (suggesting power to detect known positive control T2D-associated non-coding common variants).

## Usage Notes

The WGS and WES panels may be useful for simulation-based approaches that require individual-level genotypes and phenotypes. In this case the full list of ‘QC+’ variants (not merely those included in the T2D analysis) should be used, as the association analysis omitted very rare variants that might be useful in other settings. The WGS panel can also serve as a reference panel for genotype imputation, particularly in cases where an excess of haplotypes from T2D cases are required. Although more recent and larger efforts such as the Haplotype Reference Consortium^8^ will provide greater imputation power for most use cases, the WGS panel is not restricted based on minor allele count and includes indels and SVs.

The most valuable data from the T2D-GENES and GoT2D studies are likely the catalogues of T2D association statistics for low-frequency and common variation. These statistics may prove useful for fine mapping or functional studies of T2D GWAS signals, in which enumeration of potential causal variants is required, or for ‘reverse genetic’ approaches, in which estimates of the phenotypic effects of variants with strong molecular effects are desired. For this usage, we advise investigators to query the T2D Knowledge Portal at www.type2diabetesgenetics.org as a first step, as its goal is to provide a simple and continuously updated means to query these and other association statistics. The portal is designed specifically for queries about individual variants or those within a single gene or genomic locus, as well as variant- or gene-level analyses for which investigators desire to adjust included variants, covariates, or individuals. Users should note that data from the T2D-GENES and GoT2D studies are included in, and thus should not be combined with data from, the Exome Aggregation Consortium (exac.broadinstitute.org).

Should investigators desire access to all association statistics, genome-wide, the files at EGA should be used (as the T2D Knowledge Portal does not support bulk download of association statistics). For single variant associations, a statistic in the largest available sample size should be used. Coding variant association statistics present in both the WES panel analysis and the exome chip analysis can be safely combined via meta-analysis, as can non-coding variant association statistics present in both the WGS panel analysis and the imputation-based analysis; statistics should not, however, be combined across the non-coding and coding variant analyses. Association results from the sequence or exome chip data should not need to be filtered, but it is advisable to filter results from the imputation-based analysis according to a threshold on imputation quality (e.g., r^2^>0.3). For gene-level analyses, investigators should first use the aggregate association statistics and then dissect results by examining variant-level statistics for each variant in the mask.

The pre-computed statistics should be sufficient for most investigators. Cases where recalculation of associations may be appropriate include (a) conditional analyses, such as variant association controlling for additional individual phenotypes or genotypes at different variants, (b) association analyses with phenotypes other than T2D, and (c) novel statistical tests. In these cases, usage of the tests and inclusion of the covariates described in the methods section is recommended, and only variants and individuals present in the final ‘QC+’ analysis lists should be included.

## Additional information

**How to cite this article:** Flannick, J. *et al.* Sequence data and association statistics from 12,940 type 2 diabetes cases and controls. *Sci. Data* 4:170179 doi: 10.1038/sdata.2017.179 (2017).

**Publisher’s note:** Springer Nature remains neutral with regard to jurisdictional claims in published maps and institutional affiliations.

## Supplementary Material



Supplementary Information

## Figures and Tables

**Table 1:** Summary of studies included in WGS panel.

**Ancestry**	**Study**	**Countries of Origin**	**Num. of Cases (% female)**	**Num. of Controls (% female)**	**Total Sample Size**
European	Finland-United States Investigation of NIDDM Genetics (FUSION) Study	Finland	493 (41.5)	486 (45.2)	979
European	Kooperative Gesundheitsforschung in der Region Augsburg (KORA)	Germany	101 (44.5)	104 (66.3)	205
European	Malmö-Botnia Study	Finland, Sweden	410 (51.5)	419 (44.1)	829
European	UK Type 2 Diabetes Genetics Consortium (UKT2D)	UK	322 (46.2)	322 (82.2)	644
**Total WGS Panel**	**1,326**	**1,331**	**2,657**		
Shown are the number of individuals included in association analysis for the GoT2D whole-genome sequencing study, stratified by their study of origin. Columns from left show the ancestry of individuals in each study, the name of the study (or studies), the country of origin for the individuals, the number of cases and controls, and the total number of individuals. Reproduced from Extended Data Table 1 of Fuchsberger *et al.*^8^.					

**Table 2:** Summary of studies included in WES panel.

**Ancestry**	**Study**	**Countries of Origin**	**Num. of Cases (% female)**	**Num. of Controls (% female)**	**Total Sample Size**
African American	Jackson Heart Study (JHS)	US	500 (66.6)	526 (63.3)	1,026
African American	Wake Forest School of Medicine Study (WF)	US	518 (59.5)	530 (56.0)	1,048
East Asian	Korea Association Research Project (KARE)	Korea	526 (45.6)	561 (58.5)	1,087
East Asian	Singapore Diabetes Cohort Study; Singapore Prospective Study Program	Singapore (Chinese)	486 (52.1)	592 (61.3)	1,078
European	Ashkenazi	US, Israel	506 (47.0)	355 (56.9)	861
European	Metabolic Syndrome in Men Study (METSIM)	Finland	484 (0)	498 (0)	982
European	Finland-United States Investigation of NIDDM Genetics (FUSION)	Finland	472 (42.6)	476 (45.0)	948
European	Kooperative Gesundheitsforschung in der Region Augsburg (KORA)	Germany	97 (44.3)	90 (63.3)	187
European	UK Type 2 Diabetes Genetics Consortium (UKT2D)	UK	322 (45.7)	320 (82.8)	642
European	Malmö-Botnia Study	Finland, Sweden	478 (54.8)	443 (43.8)	921
Hispanic	San Antonio Family Heart Study (SAFHS), San Antonio Family Diabetes/ Gallbladder Study (SAFDGS), Veterans Administration Genetic Epidemiology Study (VAGES), and the Investigation of Nephropathy and Diabetes Study Family Component (SAMAFS)	US	272 (58.8)	218 (58.7)	490
Hispanic	Starr County, Texas	US	749 (59.7)	704 (71.9)	1,453
South Asian	London Life Sciences Population Study (LOLIPOP)	UK (Indian Asian)	531 (14.1)	538 (15.8)	1,069
South Asian	Singapore Indian Eye Study	Singapore (Indian Asian)	563 (44.4)	585 (49.2)	1,148
	**Total WES Panel**	**6,504**	**6,436**	**12,940**	
Shown are the number of individuals included in the GoT2D and T2D-GENES exome sequencing studies, stratified by their study of origin. Columns are as described in Table 1. Reproduced from Extended Data Table 1 of Fuchsberger *et al.*^8^.					

**Table 3:** Summary of variants in the WGS panel.

**Variant Type**	**SNV**	**Indel**	**SV**
N (%total)	25.2M (94%)	1.50M (5.6%)	8,876 (0.3%)
			
**Function**	**Coding**	**Non-coding**	
N (%total)	888K (3.3%)	25.8M (96.7%)	
			
**Frequency spectrum**	**Rare (MAF<0.5%)**	**Low frequency (0.5%<MAF<5%)**	Common (MAF>5%)
N (%total)	6.26M (23%)	4.16M (16%)	16.3M (61%)
			
**dbSNP**	**b137**	**Novel**	
N (%total)	14.6M (55%)	12.1M (45%)	
Shown are aggregate statistics on the variants the WGS panel, stratified by type (SNV, indel, or SV), function, frequency, and presence in dbSNP b137. Adapted from Extended Data Table 2 in Fuchsberger *et al.*^8^.			

**Table 4:** Summary of variant annotations in the WES panel.

**Variant annotation**	**All samples**	**African-American**	**East-Asian**	**European**	**Hispanic**	**South-Asian**
Synonymous SNV	627,630	237,430	178,232	192,282	156,231	211,218
Missense SNV	1,110,897	354,797	296,707	327,049	231,351	344,191
Start SNV	2,055	593	523	639	384	583
Nonsense SNV	26,321	7,188	6,668	8,030	4,660	7,339
Frameshift INDEL	26,901	6,605	6,159	7,515	4,155	6,609
Inframe INDEL	11,090	3,471	2,963	3,145	2,068	3,165
3′UTR SNV, INDEL	65,013	24,583	19,149	21,102	16,959	22,177
5′UTR SNV, INDEL	43,965	16,920	13,520	15,562	11,634	15,595
Intron SNV, INDEL	931,449	352,398	270,564	296,970	243,139	314,810
Essential splicing SNV, INDEL	14,286	3,648	3,454	4,108	2,301	3,744
Other splicing SNV, INDEL	128,644	45,876	35,413	38,263	30,301	41,122
Non-coding RNA SNV, INDEL	18,113	7,247	5,996	6,715	5,084	6,706
Intergenic SNV, INDEL	37,345	14,335	11,498	13,614	10,700	12,937
**All**	**3,043,709**	**1,075,091**	**850,846**	**934,994**	**718,967**	**990,196**
Shown are aggregate statistics on variants in the WES panel, stratified by predicted molecular function. Variant annotations are produced from the Variant Effect Predictor^32^. Adapted from Extended Data Table 2 in Fuchsberger *et al.*^8^.						

**Table 5:** Summary of coding variant frequencies in the WES panel.

**Variant Frequency**	**All samples**	**African-American**	**East-Asian**	**European**	**Hispanic**	**South-Asian**
Rare (MAF<0.5%)	95.79%	83.30%	90.06%	89.19%	84.56%	89.89%
*private*	*77.93%*	*53.79%*	*65.47%*	*51.80%*	*37.26%*	*61.55%*
*cosmopolitan*	*0.35%*	*1.80%*	*3.02%*	*1.88%*	*2.24%*	*1.73%*
Low frequency (0.5%<MAF<5%)	2.57%	10.36%	4.61%	5.52%	8.21%	5.10%
*private*	*0.17%*	*1.43%*	*1.10%*	*0.26%*	*0.52%*	*1.02%*
*cosmopolitan*	*0.60%*	*1.50%*	*1.54%*	*1.94%*	*2.74%*	*1.62%*
Common (MAF>5%)	1.65%	6.35%	5.33%	5.29%	7.23%	5.00%
*private*	*0.09%*	*0.00%*	*0.00%*	*0.00%*	*0.01%*	*0.00%*
*cosmopolitan*	*1.50%*	*4.35%*	*5.17%*	*4.97%*	*6.88%*	*4.86%*
Shown are aggregate frequency statistics on coding variants in the WES panel, stratified by frequency. Counts and frequencies are shown for variants specific to each ancestry, as well as overall. Private: unique to one ancestry group; Cosmopolitan: observed across all ancestry groups. Adapted from Extended Data Table 2 in Fuchsberger *et al.*^8^.						

**Table 6:** Additional cardiometabolic phenotypes measured in individuals included in the WGS and WES panels.

**Phenotype (units)**	**WGS panel**		**WES panel**					
	**Cases**		**Controls**		**Cases**		**Controls**	
	***N***	**Mean (s.d.)**	***N***	**Mean (s.d.)**	***N***	**Mean (s.d.)**	***N***	**Mean (s.d.)**
Age (yr)	1326	54.9 (9.3)	1331	64.3 (8.6)	6506	57.9 (10.1)	6434	57.9 (13.0)
Age at diagnosis (yr)	0	—	0	—	3745	48.3 (10.4)	0	—
BMI (kg/m^2^)	1326	27.6 (4.9)	1326	30.6 (5.0)	6431	28.6 (5.6)	6381	27.8 (5.8)
Weight (kg)	0	—	0	—	5067	79.0 (18.3)	5063	73.9 (18.5)
Height (cm)	1326	168.9 (9.5)	1326	166.6 (9.1)	6433	165.9 (10)	6385	165.2 (10.4)
Waist-Hip Ratio	1114	0.94 (0.08)	1224	0.91 (0.1)	0	—	0	—
Hip circumference (cm)	1114	105.1 (9.7)	1224	109 (10.5)	4454	103.1 (11.0)	4301	102.8 (11.8)
Waist circumference (cm)	1114	98.6 (13)	1224	99.1 (13.1)	4995	99.8 (14.3)	5158	94.1 (13.9)
Fasting blood glucose (mmol/l)	22	9.9 (2.8)	1330	5.2 (0.53)	2837	8.6 (3.4)	5247	5.0 (0.56)
2-hour glucose (mmol/l)	0	—	0	—	637	13.9 (4.4)	1942	6.3 (1.8)
HbA1C (%)	0	—	0	—	4403	8.4 (15.1)	3098	5.6 (0.43)
Fasting blood insulin (μIU/ml)	7	1.26 (1.1)	1070	43.9 (41.2)	1993	19.6 (26.9)	4677	17.3 (25.6)
2-hour insulin (μIU/ml)	0	—	0	—	613	51.7 (60.4)	1222	37.1 (50.4)
2-hour C-peptide (ng/ml)	0	—	0	—	52	1.7 (1.4)	34	2.1 (1.9)
GAD antibodies (nmol/l)	0	—	0	—	484	3.3 (4.5)	0	—
Total cholesterol (mmol/l)	964	5.4 (1.2)	1283	5.7 (1.0)	5530	5.1 (1.2)	5813	5.3 (1.0)
LDL (mmol/l)	809	3.3 (1.0)	1275	3.7 (0.97)	4410	3.1 (0.98)	4583	3.4 (0.93)
HDL (mmol/l)	847	1.23 (0.35)	1282	1.4 (0.41)	5395	1.2 (0.35)	5811	1.4 (0.39)
TG (mmol/l)	963	2.0 (1.8)	1282	1.4 (0.7)	5524	2.0 (1.6)	5812	1.5 (0.89)
Systolic blood pressure (mmHg)	622	142 (21.2)	904	134.4 (18.1)	5143	135.8 (20.4)	5411	130.2 (19.9)
Diastolic blood pressure (mmHg)	622	83.4 (11.2)	904	80.6 (10.3)	5143	79.1 (11.4)	5411	78.6 (11.0)
Creatinine (μmol/l)	0	—	0	—	2819	85.8 (42.9)	3189	84.4 (33.3)
Leptin (ng/ml)	0	—	0	—	559	29.4 (23.4)	658	27.3 (23.9)
Adiponectin (μg/ml)	0	—	0	—	957	6.6 (5.4)	1733	6.6 (5.1)
Diabetes medication (%)	0	—	0	—	3770	70.7%	3622	0%
Lipids medication (%)	1187	19.7%	1213	11.1%	5688	38.4%	5569	14.6%
Blood pressure medication (%)	755	50.6%	726	34.6%	4589	58.8%	4451	30.8%
For each phenotype, shown are the number of samples with the phenotype measured, the mean value of the phenotype, and its standard deviation in cases within the WGS panel, controls within the WGS panel, cases within the WES panel, and controls within the WGS panel. Some values should be used with caution, such as glycemic measurements in diabetes cases, and others should likely be adjusted prior to use, such as lipid values in individuals on lipid medications. Only the phenotypes directly available are listed in the table; some unmeasured phenotypes (such as Waist-Hip Ratio for samples in the WES panel) can be inferred from other phenotypes.								

**Table 7:** Summary of datasets.

**Participants**	**Genotyping**	**Quality control**	**Association analysis**	**Resources**	**Accession**
2,874 Europeans from the GoT2D consortium analysis	5x whole-genome sequencing, 82x exome sequencing, SNP array genotyping	Integration, phasing, individual and variant exclusions	Single variant (allele count above 3)	Sequence reads	phs000840.v1.p1
				WGS panel, individual phenotypes	phs000840.v1.p1 EGAS00001001459
				Lists of individuals and variants in association analysis, variant association statistics	EGAS00001001459
13,008 individuals from the T2D-GENES consortium analysis	82x exome sequencing	Individual and variant exclusions	Single variant, gene-level (four masks)	WES panel, individual phenotypes for all samples	EGAS00001001460
				QC+ variant list, list of individuals and variants in association analysis, variant association statistics, gene-level variant masks, gene-level association statistics	EGAS00001001460
				Sequence reads, genotypes and phenotypes (Starr County individuals)	phs001099.v1.p1
				Sequence reads, genotypes and phenotypes (JHS individuals)	phs001098.v1.p1
				Sequence reads, genotypes and phenotypes (SAMAFS individuals)	phs000849.v1.p1
				Sequence reads, genotypes and phenotypes (Singapore Chinese and Singapore Indian individuals)	phs001097.v1.p1
				Sequence reads, genotypes and phenotypes (KARE individuals)	phs001096.v1.p1
				Sequence reads, genotypes and phenotypes (Ashkenazi individuals)	phs001095.v1.p1
				Sequence reads, genotypes and phenotypes (LOLIPOP individuals)	phs001093.v1.p1
				Sequence reads, genotypes and phenotypes (METSIM individuals)	phs001100.v1.p1
				Sequence reads, genotypes and phenotypes (WFS individuals)	phs001102.v1.p1
44,414 Europeans	Imputation from WGS panel	Imputation quality	Single variant	Imputation quality scores, variant association statistics	EGAS00001001459
79,854 Europeans	Illumina exome array genotyping	Individual and variant exclusions	Single variant	Variant association statistics	EGAS00001001460
Datasets from the T2D-GENES and GoT2D studies consist of individual genotypes and phenotypes as well as statistics from genome- or exome-wide association analysis. Quality control has been performed to exclude problematic variants or individuals with problematic genotypes. Datasets are available at dbGAP and the EGA.					
