# Erratum: Sequence data and association statistics from 12,940 type 2 diabetes cases and controls

**DOI:** 10.1038/sdata.2018.2

**Published:** 2018-01-23

**Authors:** Jason Flannick, Christian Fuchsberger, Anubha Mahajan, Tanya M. Teslovich, Vineeta Agarwala, Kyle J. Gaulton, Lizz Caulkins, Ryan Koesterer, Clement Ma, Loukas Moutsianas, Davis J. McCarthy, Manuel A. Rivas, John R. B. Perry, Xueling Sim, Thomas W. Blackwell, Neil R. Robertson, N William Rayner, Pablo Cingolani, Adam E. Locke, Juan Fernandez Tajes, Heather M. Highland, Josee Dupuis, Peter S. Chines, Cecilia M. Lindgren, Christopher Hartl, Anne U. Jackson, Han Chen, Jeroen R. Huyghe, Martijn van de Bunt, Richard D. Pearson, Ashish Kumar, Martina Müller-Nurasyid, Niels Grarup, Heather M. Stringham, Eric R. Gamazon, Jaehoon Lee, Yuhui Chen, Robert A. Scott, Jennifer E. Below, Peng Chen, Jinyan Huang, Min Jin Go, Michael L. Stitzel, Dorota Pasko, Stephen C. J. Parker, Tibor V. Varga, Todd Green, Nicola L. Beer, Aaron G. Day-Williams, Teresa Ferreira, Tasha Fingerlin, Momoko Horikoshi, Cheng Hu, Iksoo Huh, Mohammad Kamran Ikram, Bong-Jo Kim, Yongkang Kim, Young Jin Kim, Min-Seok Kwon, Juyoung Lee, Selyeong Lee, Keng-Han Lin, Taylor J. Maxwell, Yoshihiko Nagai, Xu Wang, Ryan P Welch, Joon Yoon, Weihua Zhang, Nir Barzilai, Benjamin F. Voight, Bok-Ghee Han, Christopher P. Jenkinson, Teemu Kuulasmaa, Johanna Kuusisto, Alisa Manning, Maggie C. Y. Ng, Nicholette D. Palmer, Beverley Balkau, Alena Stančáková, Hanna E. Abboud, Heiner Boeing, Vilmantas Giedraitis, Dorairaj Prabhakaran, Omri Gottesman, James Scott, Jason Carey, Phoenix Kwan, George Grant, Joshua D. Smith, Benjamin M. Neale, Shaun Purcell, Adam S. Butterworth, Joanna M. M. Howson, Heung Man Lee, Yingchang Lu, Soo-Heon Kwak, Wei Zhao, John Danesh, Vincent K. L. Lam, Kyong Soo Park, Danish Saleheen, Wing Yee So, Claudia H. T. Tam, Uzma Afzal, David Aguilar, Rector Arya, Tin Aung, Edmund Chan, Carmen Navarro, Ching-Yu Cheng, Domenico Palli, Adolfo Correa, Joanne E. Curran, Dennis Rybin, Vidya S. Farook, Sharon P. Fowler, Barry I. Freedman, Michael Griswold, Daniel Esten Hale, Pamela J. Hicks, Chiea-Chuen Khor, Satish Kumar, Benjamin Lehne, Dorothée Thuillier, Wei Yen Lim, Jianjun Liu, Marie Loh, Solomon K. Musani, Sobha Puppala, William R. Scott, Loïc Yengo, Sian-Tsung Tan, Herman A. Taylor, Farook Thameem, Gregory Wilson, Tien Yin Wong, Pål Rasmus Njølstad, Jonathan C. Levy, Massimo Mangino, Lori L. Bonnycastle, Thomas Schwarzmayr, João Fadista, Gabriela L. Surdulescu, Christian Herder, Christopher J. Groves, Thomas Wieland, Jette Bork-Jensen, Ivan Brandslund, Cramer Christensen, Heikki A. Koistinen, Alex S .F. Doney, Leena Kinnunen, Tõnu Esko, Andrew J. Farmer, Liisa Hakaste, Dylan Hodgkiss, Jasmina Kravic, Valeri Lyssenko, Mette Hollensted, Marit E. Jørgensen, Torben Jørgensen, Claes Ladenvall, Johanne Marie Justesen, Annemari Käräjämäki, Jennifer Kriebel, Wolfgang Rathmann, Lars Lannfelt, Torsten Lauritzen, Narisu Narisu, Allan Linneberg, Olle Melander, Lili Milani, Matt Neville, Marju Orho-Melander, Lu Qi, Qibin Qi, Michael Roden, Olov Rolandsson, Amy Swift, Anders H. Rosengren, Kathleen Stirrups, Andrew R. Wood, Evelin Mihailov, Christine Blancher, Mauricio O. Carneiro, Jared Maguire, Ryan Poplin, Khalid Shakir, Timothy Fennell, Mark DePristo, Martin Hrabé de Angelis, Panos Deloukas, Anette P. Gjesing, Goo Jun, Peter Nilsson, Jacquelyn Murphy, Robert Onofrio, Barbara Thorand, Torben Hansen, Christa Meisinger, Frank B. Hu, Bo Isomaa, Fredrik Karpe, Liming Liang, Annette Peters, Cornelia Huth, Stephen P. O'Rahilly, Colin N. A. Palmer, Oluf Pedersen, Rainer Rauramaa, Jaakko Tuomilehto, Veikko Salomaa, Richard M. Watanabe, Ann-Christine Syvänen, Richard N. Bergman, Dwaipayan Bharadwaj, Erwin P. Bottinger, Yoon Shin Cho, Giriraj R. Chandak, Juliana C. N. Chan, Kee Seng Chia, Mark J. Daly, Shah B. Ebrahim, Claudia Langenberg, Paul Elliott, Kathleen A. Jablonski, Donna M. Lehman, Weiping Jia, Ronald C. W. Ma, Toni I. Pollin, Manjinder Sandhu, Nikhil Tandon, Philippe Froguel, Inês Barroso, Yik Ying Teo, Eleftheria Zeggini, Ruth J. F. Loos, Kerrin S Small, Janina S. Ried, Ralph A. DeFronzo, Harald Grallert, Benjamin Glaser, Andres Metspalu, Nicholas J. Wareham, Mark Walker, Eric Banks, Christian Gieger, Erik Ingelsson, Hae Kyung Im, Thomas Illig, Paul W. Franks, Gemma Buck, Joseph Trakalo, David Buck, Inga Prokopenko, Reedik Mägi, Lars Lind, Yossi Farjoun, Katharine R. Owen, Anna L. Gloyn, Konstantin Strauch, Tiinamaija Tuomi, Jaspal Singh Kooner, Jong-Young Lee, Taesung Park, Peter Donnelly, Andrew D. Morris, Andrew T. Hattersley, Donald W. Bowden, Francis S. Collins, Gil Atzmon, John C. Chambers, Timothy D. Spector, Markku Laakso, Tim M. Strom, Graeme I. Bell, John Blangero, Ravindranath Duggirala, E Shyong Tai, Gilean McVean, Craig L. Hanis, James G. Wilson, Mark Seielstad, Timothy M. Frayling, James B. Meigs, Nancy J. Cox, Rob Sladek, Eric S. Lander, Stacey Gabriel, Karen L. Mohlke, Thomas Meitinger, Leif Groop, Goncalo Abecasis, Laura J. Scott, Andrew P. Morris, Hyun Min Kang, David Altshuler, Noël P. Burtt, Jose C. Florez, Michael Boehnke, Mark I. McCarthy

*Scientific Data* 4:170179 doi: 10.1038/sdata.2017.179 (2017); Published 19 December 2017; Updated: 23 January 2018.

In both the HTML and PDF versions of this Data Descriptor, the author name Jason Flannick was incorrectly listed as Flannick Jason.

