# Supplementary material for: Sequence data and association statistics from 12,940 type 2 diabetes cases and controls
Source: Sci Data. 2017 Dec 19;4:170179. doi: 10.1038/sdata.2017.179 (PMC5735917; doi:10.1038/sdata.2017.179)
Supplement: Supplementary Information [file sdata2017179-s2.pdf]

<u>**Supplementary Information**</u>

Acknowledgements

Albert Einstein College of Medicine, USA

Funders of this work:
• The American Federation for Aging Research
• The Einstein Glenn Center
• National Institute on Aging (PO1AG027734, R01AG046949, 1R01AG042188, P30AG038072)

Broad Institute, USA

Funders of this work:
• NHGRI ('Large Scale Sequencing and Analysis of Genomes' U54HG003067)
• NIDDK ('Multi-ethnic Study of Type 2 Diabetes Genes' U01DK085526)
• NIH ('Low-Pass Sequencing and High Density SNP Genotyping in Type 2 Diabetes'1RC2DK088389)

Personal support:
• Vineeta Agarwala: National Institute of General Medical Sciences award (T32GM007753)

Center for Genome Science, National Institute of Health, Republic of Korea
Funders of this work:

• Korea National Institute of Health (2012-N73002-00)
• Korea National Institute of Health and Korea Centers for Disease Control and Prevention (4845–301)

Other acknowledgements:

• This study was provided with biospecimens and data from the Korean Genome Analysis Project (4845-301), the Korean Genome and Epidemiology Study (4851-302), and the Korea Biobank Project (4851-307, KBP-2013-11 and KBP-2014-68) that were supported by the Korea Centers for Disease Control and Prevention, Republic of Korea.

The Chinese University of Hong Kong

Funders of this work:
• The Focused Investment Scheme of the Chinese University of Hong Kong
• The Hong Kong Foundation for Research and Development in Diabetes established under the auspices of the Chinese University of Hong Kong
• Hong Kong Governments Research Grant Committee Central Allocation Scheme (CUHK1/04C)
• The Innovation and Technology Fund (ITS/487/09FP, ITS/130/11)
• The Hong Kong Research Grants Council Theme-based Research Scheme (T12-402/13N)

German Diabetes Center, Germany

Funders of this work:
• Ministry of Science and Research of the State of North Rhine-Westphalia (MIWF NRW)
• German Federal Ministry of Health (BMG)
• Grant from the German Federal Ministry of Education and Research (BMBF) to the German Center for Diabetes Research (DZD)

GoT2D consortium

Funders of this work:
• National Institutes of Health ('Low-Pass Sequencing and High-Density SNP Genotyping for Type 2 Diabetes' RC2DK088389)
The German Center for Diabetes Research (DZD)

Hallym University Chuncheon, Republic of Korea

Funders of this work:
• National Research Foundation of Korea (NRF-2012R1A2A1A03006155)

Helmholtz Zentrum München—German Research Center for Environmental Health, Germany

Funders of this work:

• The German Center for Diabetes Research (DZD)
• Helmholtz Zentrum München (German Research Center for Environmental Health),which is supported by the German Federal Ministry of Education and Research (BMBF)and by the State of Bavaria
• The Munich Center of Health Sciences (MC-Health), Ludwig-Maximilians-Universität, aspart of LMUinnovativ

Other acknowledgements:

• The KORA research platform (KORA, Cooperative Research in the Region of Augsburg) was initiated and financed by the Helmholtz Zentrum München—German Research Center for Environmental Health, which is funded by the German Federal Ministry of Education and Research and by the State of Bavaria. Furthermore, KORA research was supported within the Munich Center of Health Sciences (MC Health), Ludwig- Maximilians-Universität, as part of LMUinnovativ.

Imperial College London, UK

Funders of this work:

• Action on Hearing Loss (G51)
• The British Heart Foundation (SP/04/002)
• European Union FP7 (EpiMigrant, 279143)
• Medical Research Council (G0601966, G0700931)
• Medical Research Council (MR/L01632X/1 UK MEDical BIOinformatics partnership: aggregation, integration, visualization, and analysis of large, complex data
• MRC-PHE Centre for Environment and Health
• The National Institute for Health Research (NIHR) (RP-PG-0407-10371)
• NIHR Biomedical Research Centre at Imperial College Health Care NHS Trust
• NIHR Health Protection Research Unit on Health Impact of Environmental Hazards
• The Wellcome Trust (084723)

Personal Support

• Paul Elliot: NIHR Senior Investigator

Other acknowledgements:

• The LOLIPOP study is supported by the National Institute for Health Research (NIHR) Comprehensive Biomedical Research Centre Imperial College Healthcare NHS Trust. The work was carried out in part at the NIHR/Wellcome Trust Imperial Clinical Research Facility. We thank the participants and research staff who made the study possible.

The Jackson Heart Study, USA

Funders of this work:

• National Heart, Lung, and Blood Institute and the National Institute on Minority Health a nd Health Disparities (HHSN268201300046C, HHSN268201300047C, HHSN268201300048C, HHSN268201300049C, HHSN268201300050C)

The Jackson Laboratory for Genomic Medicine, USA

Funders of this work:

• National Institutes of Health (R00DK092251)

King’s College, London

Funders of this work:

• European Community’s Seventh Framework Programme (FP7/2007-2013)
• National Institute for Health Research (NIHR)- funded BioResource, Clinical Research Facility and Biomedical Research Centre based at Guy's and St Thomas' NHS FoundationTrust in partnership with King's College London
• The Wellcome Trust

Lund University, Sweden

Funders of this work:

• The Academy of Finland
• European Research Council Advanced Research Grant
• The Folkhälsan Research Foundation
• Novo Nordisk
• The Påhlssons Foundation
• The Sigrid Juselius Foundation
• The Skåne Regional Health Authority
• The Swedish Heart-Lung Foundation
• Swedish Research Council (Linné and Strategic Research Grant)

Massachusetts General Hospital, USA

Funders of this work:

• National Institutes of Health (U01DK085526)

Personal support:

• Jose Florez: MGH Research Scholar
• James B Meigs: National Institutes of Health (K24DK080140)

McGill University, Canada

Funders of this work:

• The Canadian Institutes of Health Research

Personal support:

• Rob Sladek: Chercheur Boursier award from the Fonds de la Recherche en Santé du Québec; New Investigator Award from the Canadian Institutes of Health Research

National Institute for Health and Welfare, Helsinki, Finland

Funders of this work:

• The Academy of Finland (139635)
• The Finnish Foundation for Cardiovascular Research

National University of Singapore

Funders of this work:

• Biomedical Research Council (BMRC) Individual Research Grant
• National Medical Research Council (NMRC) Individual Research Grant
• NMRC Centre Grant

Personal support:

• Ching-Yu Cheng: NMRC Clinician Scientist award
• E Shyong Tai: NMRC Clinician Scientist award
• YY Teo: National Research Foundation Fellowship
• TY Wong: NMRC Singapore Translational Research Investigator award

Seoul National University, Republic of Korea

Funders of this work:

• Korea Health Technology R&D Project through the Korea Health Industry Development Institute (KHIDI), funded by the Ministry of Health & Welfare, Republic of Korea (HI14C0060)
• National Research Foundation of Korea (NRF) grants (2012R1A3A2026438, 2013M3A9C4078158, 2008-0062618)

T2D-GENES and GoT2D consortium

Funders of this work:

• National Institutes of Health ('Multiethnic Study of Type 2 Diabetes Genes' U01s DK085526, DK085501, DK085524, DK085545, DK085584; 'Low-Pass Sequencing and High-Density SNP Genotyping for Type 2 Diabetes' DK088389)
• The German Center for Diabetes Research (DZD)

University of Bergen, Norway

Funders of this work:

• European Research Council (ERC-2011-ADG_20110310 #293574)
• The KG Jebsen Foundation
• Research Council of Norway
• University of Bergen
• The Western Norway Regional Health Authority (Helse Vest)

University of Chicago, USA

Funders of this work:

• National Institute of Diabetes and Digestive and Kidney Diseases (NIDDK) (P30 DK20595)
• National Institutes of Health (R01MH101820, P60DK20595, U01DK085501, R01HL102830, U01HG005773, R01MH090937)

Personal support:

Hae Kyung Im: National Institutes of Mental Health (NIMH) (R01 MH107666)

Other acknowledgements:

• The content of this manuscript is solely the responsibility of the authors and does not necessarily represent the official views of the National Cancer Institute or the National Institutes of Health.

University of Cambridge, UK

Funders of this work:

• UK Medical Research Council (G0800270)
• British Heart Foundation (SP/09/002)
• European Research Council (268834)
• European Union Framework Programme 7 (HEALTH-F2-2012-279233)
• National Institute for Health Research, Cambridge Biomedical Research Centre
• Merck
• Pfizer

Personal support:

• John Danesh: British Heart Foundation Professorship, NIHR Senior Investigator, European Research Council Senior Investigator.

University of Copenhagen

Funders of this work:

• The Novo Nordisk Foundation Center for Basic Metabolic Research, an independent Research Center at the University of Copenhagen partially funded by an unrestricted donation from the Novo Nordisk Foundation

University of Eastern Finland

Funders of this work:
• The Academy of Finland
• Strategic Research Funding from the University of Eastern Finland, Kuopio, Finland

University of Helsinki, Finland

Personal support:

• Heikki A Koistinen: Academy of Finland Clinical Researcher

University of Michigan, USA

Funders of this work:
• National Institutes of Health (R01DK062370, R01DK098032, RC2DK088389)

University of North Carolina, USA

Funders of this work:
• National Institutes of Health (DK072193, DK093757)

University of Oxford, UK

Funders of this work:
• The European Commission (ENGAGE: HEALTH-F4-2007-201413; Marie-Curie Fellowship PIEFGA-2012-329156)
• MRC (G0601261, G0900747-91070)
• National Institutes of Health (RC2-DK088389, DK085545, DK098032)
• Wellcome Trust (064890, 083948, 085475, 086596, 090367, 090532, 092447, 095101,095552, 098017, 098381, 100956)

Personal support:

• Peter Donnelly: Wellcome Trust Senior Investigator
• Andrew Farmer: NIHR Senior Investigator
• Anna L Gloyn: Wellcome Trust Senior Fellow in Basic Biomedical Science
• Fredrik Karpe: NIHR Oxford Biomedical Research Centre; NIHR National Bioresource
• Cecilia Lindgren: Wellcome Trust Intermediate Research Fellow; Li Ka Shing Foundation
• Davis J McCarthy: General Sir John Monash Scholarship
• Mark I McCarthy: Wellcome Trust Senior Investigator; NIHR Senior Investigator
• Gilean McVean: Wellcome Trust Senior Investigator
• Andrew P Morris: Wellcome Trust Senior Fellow in Basic Biomedical Science
• Katharine R Owen: NIHR Clinician Scientist
• John R B Perry: Sir Henry Wellcome Postdoctoral Fellowship
• Manuel A Rivas: NDM Prize Studentship—Clarendon Award
• Juan Fernandez Tajes: Marie-Curie Fellow
• Martijn van de Bunt: NDM Prize Studentship

Other acknowledgements:

• We thank the High-Throughput Genomics Group at the Wellcome Trust Centre for Human Genetics for the generation of array and sequencing data.

The University of Pennsylvania

Personal support:

• Benjamin F Voight: National Institutes of Health (R01DK101478)

The University of Texas Health Science Center at Houston, USA

Funders of this work:
• National Institutes of Health (U01DK085501, R01HL102830, R01DK073541)

Personal support:

• Heather Highland: NHLBI training grant (T32 HL007055)

Uppsala University

Funders of this work:

• The European Research Council
• The Knut och Alice Wallenberg Foundation
• The Swedish Heart-Lung Foundation (20140422)
• The Swedish Diabetes Foundation (2013-024)
• The Swedish Research Council (2012-1397)
• Uppsala University

Wake Forest School of Medicine, USA

Funders of this work:

• National Institutes of Health (R01DK066358)

Wellcome Trust Sanger Institute, UK

Funders of this work:

• National Institute for Health Research
• The Wellcome Trust (098051)

Other acknowledgements:

• Panos Deloukas's work forms part of the research themes contributing to the translational research portfolio of Barts Cardiovascular Biomedical Research Unit, which is supported and funded by the National Institute for Health Research.

Studies

EGCUT received financing from European Regional Development Fund, road-map grant no.3.2.0304.11-0312 and grant 'Center of Excellence in Genomics (EXCEGEN). EGCUT studies were covered also by targeted financing from Estonian Government (IUT24-6, IUT20-60) and CTG grant (SP1GVARENG) from Development Fund of the University of Tartu.

PIVUS was supported by grants from Uppsala University, Swedish Research Council (2012- 1397), Swedish Heart-Lung Foundation (20140422), Knut och Alice Wallenberg Foundation, European Research Council, Swedish Diabetes Foundation (2013-024).

The Botnia study has been financially supported by grants from the Sigrid Juselius Foundation, Folkhälsan Research Foundation, Nordic Center of Excellence in Disease Genetics, an EU grant (EXGENESIS), Signe and Ane Gyllenberg Foundation, Swedish Cultural Foundation in Finland, Finnish Diabetes Research Foundation, Foundation for Life and Health in Finland, Finnish Medical Society, Paavo Nurmi Foundation, Helsinki University Central Hospital Research Foundation, Perklén Foundation, Ollqvist Foundation, Närpes Health Care Foundation and Ahokas Foundation. The study has also been supported by the Ministry of Education in Finland, Municipal Heath Care Center and Hospital in Jakobstad and Health Care Centers in Vasa, Närpes and Korsholm.

The Mount Sinai BioMe Biobank has been financially supported The Andrea and Charles Bronfman Philanthropies.

Other

This study also utilized the high-performance computational capabilities of the Biowulf Linux cluster at the National Institutes of Health, Bethesda, Md. (http://biowulf.nih.gov).

Current addresses:

• David Altshuler: Vertex Pharmaceuticals, Boston, MA, USA

• Rector Arya, Ravindranath Duggirala, Vidya S Farook, Christopher P Jenkinson: South Texas Diabetes and Obesity Institute, Edinburg Regional Academic Health Center, University of Texas

• Health Science Center at San Antonio/University of Texas Rio Grande Valley, Edinburg, TX 78541

• Dwaipayan Bharadwaj: School of Biotechnology, Jawaharlal Nehru University, New Delhi

• Han Chen: The University of Texas Health Science Center at Houston, Houston, Texas, USA

• Nancy J Cox: Vanderbilt Genetics Institute, Vanderbilt University Medical Center, Nashville, TN, USA

• Mark DePristo: Google Inc., Mountain View, CA, USA

• Christian Fuchsberger: Division of Genetic Epidemiology, Department of Medical Genetics, Molecular and Clinical Pharmacology, Medical University of Innsbruck, Innsbruck, Austria; Center for Biomedicine, European Academy of Bolzano/Bozen (EURAC), affiliated with the University of Lübeck, Bolzano, Italy.

• Eric Gamazon: Vanderbilt Genetics Institute, Vanderbilt University Medical Center, Nashville, TN, USA

• Kyle Gaulton: Department of Pediatrics, University of California San Diego, San Diego, CA, USA

• Heather Highland: Department of Epidemiology, University of North Carolina at Chapel Hill, Chapel Hill, NC, USA

• Jinyan Huang: State Key Laboratory of Medical Genomics, Shanghai Institute of Hematology, Rui Jin Hospital, Shanghai Jiao Tong University School of Medicine, Shanghai 200025, China

• Jeroen Huyghe: Public Health Sciences Division, Fred Hutchinson Cancer Research Center, Seattle, Washington 98109, USA

• Erik Ingelsson: Department of Medicine, Division of Cardiovascular Medicine, Stanford University School of Medicine, Stanford, California, USA

• Ashish Kumar: Institute of Environmental Medicine, Karolinska Institutet, Stockholm, Sweden; Chronic Disease Epidemiology, Swiss Tropical and Public Health Institute, University of Basel, Basel, Switzerland

• Claes Ladenvall: Dept of Immunology, Genetics and Pathology, Uppsala University, Sweden

• Cecilia M Lindgren: The Big Data Institute at the Li Ka Shing Centre for Health Information and Discovery, University of Oxford, Oxford OX3 7BN, UK

• Jong-Young Lee:NGS Division, Theragen Etex Bio Institute, 2nd Floor, B-dong AICT Bldg., 145 Gwanggyo-ro, Yeongtong-gu, Suwon-si, Gyeonggi-do, Republic of Korea, 16229

• Adam Locke: McDonnell Genome Institute, Washington University School of Medicine, St Louis, MO, USA

• Clement Ma: Dana-Farber/Boston Children's Cancer and Blood Disorders Center, Boston, MA, USA

• Loukas Moutsianas: Department of Human Genetics, Wellcome Trust Sanger Institute, Hinxton, Cambridgeshire, UK

• Xueling Sim: Saw Swee Hock School of Public Health, National University of Singapore, National University Health System, Singapore

•Herman A Taylor Jr: Morehouse School of Medicine, Morehouse College, Atlanta, GA, USA

•Farook Thameem: Department of Biochemistry, Faculty of Medicine, Kuwait University, Kuwait

•Sobha Puppala: Department of Internal Medicine-Section of Molecular Medicine, Wake Forest School of Medicine, Winston-Salem, North Carolina, USA

Author Contributions

The following individuals contributed to analysis and deposition of data for the data descriptor. J.F., C.F., J.C.F., M.I.M, and M.B. wrote the paper. J.F., L.C., R.K., C.F., and A.M. deposited data. N.P.B., D.A., J.C.F., M.I.M., and M.B. oversaw generation and deposition of the data.

Additionally, the following individuals contributed to the primary experiment that generated and analyzed the data. Sample Collection And Phenotyping (WholeGenomes): L.L.B., J. Fadista, C. Herder, C.J.G., H.A.K., L.K., J. Kravic, V.L., C. Ladenvall, W.R., N.N., M.R., A. Swift, P.N., B.T., C. Meisinger, A. P., C. Huth, J. Tuomilehto, R.M.W., R.N.B., K.S.S., C.G., K.R.O., K. Strauch, T.T., A.T.H., F.S.C., T.D.S., T. M.F., N.P.B., K.L.M., L.G., M.B., D. Altshuler, M.I.M.

Sample Collection And Phenotyping (GWAS Imputation): C.F., H.C., M.M.-N., R.A.S., B.B., H.B., V. G., O.G., P.K., Y.L., C.N., D. Palli, D.R., D.T., Y.T.v.d.S., C. Ladenvall, E.M., A.-C.S., E.P.B., C. Langenberg, P. Froguel, R.J.F.L., A. Metspalu, N.J.W., E.I., T.I., P.W.F., R.M., L. Lind, J.B.M., L.J.S., M.B., D. Altshuler, M.I.M.

Sample Collection And Phenotyping (Whole Exomes): J.E.B., N.B., B.-G.H., C.P.J., T.K., J. Kuusisto, M. C.Y.N., N.D.P., A. Stančáková, H.E.A., U.A., D. Aguilar, R.A., T.A., E.C., C.-Y.C., A.C., J.E.C., V.S.F., S.P. F., B.I.F., M.G., D.E.H., P.J.H., C.-C.K., S.K., B.L., W.Y.L., J. Liu, M. Loh, S.K.M., S. Puppala, W.R.S., S.-T. T., H.A.T.Jr, F.T., G.W.Sr, T.Y.W., J.C.L., M.M., L.L.B., J. Fadista, G.L.S., C.J.G., L.K., D.H., J. Kravic, C. Ladenvall, N.N., A. Swift, P.N., S.P.O'R., J. Tuomilehto, Y.S.C., K.S.C., D.M.L., T.I.P., K.S.S., R.A.D., B.G., M.W., J.S.K., J.-Y.L., A.T.H., D.W.B., G. Atzmon, J.C.C., T.D.S., M. Laakso, G.I.B., J.B., R.D., E.S.T., C.L. H., J.G.W., T.M.F., N.J.C., L.G., M.B., D. Altshuler, M.I.M.

Sample Collection And Phenotyping (Exome Array & Replication): A. Mahajan, N.R.R., N.W.R., N.G., R.A.S., J.H., D. Pasko, T.V.V., S.-H.K., K.S.P., J.C.L., M.M., G.L.S., C.J.G., J.B.-J., I. Brandslund, C.C., A.S. F.D., T.E., A.J.F., L.H., D.H., J. Kravic, M. Hollensted, M.E.J., T.J., C. Ladenvall, J.M.J., A. Käräjämäki, L. Lannfelt, T.L., A.L., O.M., L. Milani, M.N., M.O.-M., L.Q., Q.Q., M.R., O.R., A.H.R., K. Stirrups, A.R.W., E.M., M.H.d.A., P. Deloukas, B.T., T.H., C. Meisinger, F.B.H., B.I., F.K., L. Liang, A.P., S.P.O'R., C.N.A.P., O.P., R.R., V.S., A.-C.S., R.N.B., C. Langenberg, K.S.S., A. Metspalu, N.J.W., M.W., C.G., E.I., T.I., P.W.F., R.M., L. Lind, K.R.O., K. Strauch, T.T., A.D.M., A.T.H., F.S.C., T.D.S., T.M.F., L.G., A.P.M., M.I.M.
Design And Study Supervision: C.F., T. Fingerlin, C. Hu, C.P.J., H.E.A., D. Prabhakaran, J.S., P.R.N., M. H.d.A., T.H., O.P., J. Tuomilehto, R.M.W., D. Bharadwaj, Y.S.C., G.R.C., J.C.N.C., K.S.C., M.J.D., S.B.E., P.E., K.A.J., D.M.L., W.J., R.C.W.M., T.I.P., M. Sandhu, N.T., P. Froguel, I. Barroso, Y.Y.T., E.Z., R.A.D., B.G., I.P., A.L.G., J.S.K., J.-Y.L., T.P., P. Donnelly, A.D.M., A.T.H., D.W.B., G. Atzmon, J.C.C., M. Laakso, T.M.S., G.I.B., J.B., R.D., E.S.T., G.M., C.L.H., J.G.W., M. Seielstad, T.M.F., J.B.M., N.J.C., R.S., E.S.L., N.P. B., K.L.M., T.M., L.G., G. Abecasis, J.C.F., L.J.S., A.P.M., M.B., D. Altshuler, M.I.M.
Data Production: J. Flannick, K.J.G., M. Hollensted, J.M.J., C.B., J. Maguire, R.P., K. Shakir, T. Fennell, M.D., J. Murphy, R.O., J.S.R., H.G., E.B., G.B., J. Trakalo, D. Buck, Y.F., T.M.S., E.S.L., S.G., N.P.B., T.M.
Variant Calling And Panel Generation: C.F., J. Flannick, A. Mahajan, K.J.G., M.A.R., X.S., N.R.R., N.W. R., P.S.C., C. Hartl, A.U.J., J.R.H., R.D.P., A. Kumar, M.M.-N., N.G., H.M.S., M.L.S., S.C.J.P., J.C., G.G., J. D.S., B.M.N., S. Purcell, T.S., T.W., J.B.-J., J. Kriebel, M.O.C., J. Maguire, R.P., K. Shakir, M.D., A.P.G., G. J., J. Murphy, R.O., E.B., Y.F., T.M.S., N.P.B., T.M., H.M.K., M.B., M.I.M.

Statistical Analysis: C.F., J. Flannick, T.M.T., A. Mahajan, V.A., K.J.G., C. Ma, L. Moutsianas, D.J.M., M. A.R., J.R.B.P., X.S., T.W.B., N.R.R., N.W.R., P. Cingolani, A.E.L., J.F.T., H.M.H., J. Dupuis, P.S.C., C.M.L., C. Hartl, A.U.J., H.C., J.R.H., M.v.d.B., R.D.P., A. Kumar, M.M.-N., N.G., H.M.S., E.R.G., Jaehoon Lee, Y. C., R.A.S., J.E.B., P. Chen, J.H., M.J.G., D. Pasko, T.V.V., T.G., N.L.B., A.G.D.-W., T. Ferreira, M. Horikoshi, I.H., M.K.I., B.-J.K., Y.K., Y.J.K., M.-S.K., Juyoung Lee, S.L., K.-H.L., T.J.M., Y.N., X.W., R.P. W., J.Y., W. Zhang, N.B., B.F.V., B.-G.H., T.K., J. Kuusisto, A. Manning, M.C.Y.N., N.D.P., B.B., A. Stančáková, A.S.B., J.M.M.H., H.M.L., Y.L., W. Zhao, J. Danesh, V.K.L.L., D.S., W.Y.S., C.H.T.T., L.Y., C. Ladenvall, J.C.N.C., C. Langenberg, R.C.W.M., T.I.P., R.J.F.L., N.J.W., E.I., H.K.I., P.W.F., I.P., R.M., K. Strauch, J.B.M., R.S., N.P.B., K.L.M., G. Abecasis, L.J.S., A.P.M., H.M.K., M.B., D. Altshuler, M.I.M.
Drafting Of Manuscript: C.F., J. Flannick, T.M.T., A. Mahajan, V.A., K.J.G., L. Moutsianas, D.J.M., M.A. R., J.R.B.P., P. Cingolani, N.J.C., R.S., N.P.B., L.J.S., A.P.M., H.M.K., M.B., D. Altshuler, M.I.M.

Project Leadership: M.B., D. Altshuler, M.I.M.
